# Metal-Glycerates and Their Derivatives: An Emerging Platform for Supercapacitors

**DOI:** 10.3390/molecules30244735

**Published:** 2025-12-11

**Authors:** Yan Zhou, Qingjie Li, Mayao Li, Zhuo Zhao, Junxi Shen, Jiaxing Feng, Keyi Zheng, Ziquan Yang, Huiyang Xu, Jiaqi Chen, Shengcheng Pan, Min Zhang, Fen Qiao, Zhen Wu, Xinlei Wang

**Affiliations:** 1 School of Energy and Power Engineering, Jiangsu University, Zhenjiang 212013, China; 2 Research Center of Fluid Machinery Engineering and Technology, Jiangsu University, Zhenjiang 212013, China; 3 School of Photoelectric Engineering, Changzhou Institute of Technology, Changzhou 213032, China; 4 School of Materials Science and Engineering, Nanjing Institute of Technology, Nanjing 211167, China; 5 College of Chemical and Material Engineering, Quzhou University, Quzhou 324000, China

**Keywords:** metal-glycerates, derivatives, electrode materials, supercapacitors

## Abstract

Supercapacitors are widely studied for their high energy density, low cost, and exceptional cycling durability. However, the decisive factor in determining the performance of supercapacitors is the electrode material. Among emerging materials, metal glycerates stand out as tunable organic-inorganic hybrids with well-controlled structures. Yet, progress in tailoring metal glycerates for supercapacitors has not been organized or consolidated into a coherent framework. Herein, we systematically summarize recent advances in the synthesis, structural evolution, and electrochemical applications of metal glycerates and their derivatives (including hydroxides, oxides, sulfides, phosphides, selenides, and composites) as electrodes for supercapacitors, emphasizing the intrinsic structure-performance correlations. Finally, the key challenges and future prospects, covering controlled synthesis, interfacial stability, mechanistic insight, and device-level integration, are discussed to guide the rational design of next-generation MG-based materials for high-performance, sustainable supercapacitor technologies.

## 1. Introduction

The rapid growth of global population, along with accelerated industrialization and economic expansion, has placed enormous strain on both energy resources and the environment [1,2,3,4,5,6,7]. Currently, fossil fuels (such as coal, oil, and natural gas) remain the dominant energy sources, yet they are nonrenewable and are being rapidly consumed, releasing substantial amounts of greenhouse gases and hazardous pollutants that severely damage the environment [8,9,10,11,12,13,14]. In response to these critical issues, the global energy landscape is undergoing a profound transformation, marked by a surge in the development and deployment of renewable energy sources such as solar, wind, and hydrogen energy [15,16,17,18,19,20,21]. Despite their environmental benefits and theoretical abundance, these renewable resources are inherently intermittent and unevenly distributed, which poses serious challenges to ensuring a stable and continuous energy supply on a large scale [22,23,24,25,26]. Consequently, converting renewable energy into electricity and storing it for reliable use is therefore considered a promising strategy to overcome these limitations [27,28]. This underscores the urgent need for advanced, low-cost, and multifunctional electrochemical energy storage (EES) devices that can effectively bridge the gap between energy generation and utilization [29,30,31,32].

Among various EES technologies, supercapacitors (SCs) have emerged as promising devices that bridge the performance gap between conventional capacitors and batteries due to their rapid charge–discharge capability, high power density, long cycle life, and environmental friendliness [33,34,35]. Based on their energy storage mechanisms, SCs are primarily classified into three categories: electric double-layer capacitors (EDLCs), pseudocapacitors (PCs), and hybrid supercapacitors (HSCs) [36,37]. EDLCs store energy through electrostatic ion adsorption at the electrode-electrolyte interface, without involving Faradaic reactions [38,39,40]. Carbon-based materials, such as activated carbon, graphene, and carbon nanotubes, are commonly used as EDLC electrodes owing to their large specific surface area, high electrical conductivity, and adjustable pore structures [40,41,42,43]. In contrast, PCs store charge via fast and reversible Faradaic redox reactions occurring at or near the surface of electrode materials, typically transition metal oxides or conducting polymers, which offer higher energy density but often suffer from structural instability and limited cycle life [44,45,46]. HSCs, meanwhile, integrate the advantages of both EDLCs and PCs by employing an asymmetric configuration consisting of a capacitive carbon-based electrode and a battery-type Faradaic electrode [47]. This combination allows HSCs to achieve enhanced energy density without sacrificing the characteristic high power output and cycle stability of EDLCs [48].

Due to their advantageous characteristics, SCs have been implemented in a variety of emerging applications, including hybrid electric vehicles, wearable electronics, and smart grids [37]. Nevertheless, their practical deployment is still constrained by their intrinsically low energy density [49,50]. Structurally, SCs typically comprise electrode materials, current collectors, separators, and electrolytes, among which the electrode materials play a pivotal role in determining electrochemical performance of the devices [39,51]. The energy density of SCs is primarily governed by the specific capacitance and operational voltage of the electrode materials, highlighting the critical need for advanced materials with optimized electrochemical properties and broader voltage windows [52,53].

In this context, metal-glycerate (MG)-based materials have garnered considerable attention as promising electrode materials for SCs due to their unique combination of inorganic and organic characteristics [54,55,56]. MGs are composed of glycerate anions and metal ions and are typically regarded as inorganic polymers that integrate the structural and chemical features of both organic and inorganic components, similar to metal–organic frameworks (MOFs) [57,58,59,60,61,62,63]. The coordination between polyhydroxy ligands and metal centers imparts MGs with notable structural stability, tunable composition, and electrochemical activity [64,65]. In addition to their direct application in SCs, MGs serve as versatile precursors or templates for fabricating advanced electrode materials [66,67,68]. For example, MGs can be converted into various metal-based compounds, including oxides, sulfides, phosphide, and other derivatives, through thermal treatment processes such as oxidation, sulfuration, or phosphorization [69,70,71]. The diversity of MGs and their derivatives largely stems from the wide selection of transition metals (e.g., Co, Ni, Fe, Mn, Mo, and V) that can be used as metal centers [50,72,73,74,75,76,77]. These ions not only construct the coordination framework but also impart rich physicochemical properties essential for energy storage applications.

Through continuous efforts in tailoring MGs and their derivatives to meet the specific requirements of SC systems, researchers have successfully developed a range of high-performance SCs. However, despite significant progress in the design and synthesis of these materials for SC applications, comprehensive reviews that systematically summarize recent advances and critically analyze the underlying structure-property relationships remain scarce. Moreover, unlike previous reviews that either take a broad view of various energy-related applications or concentrate mainly on electrocatalytic water splitting, this review focuses exclusively on metal-glycerates and their derivatives for supercapacitor applications, offering a comprehensive and in-depth analysis to better inform and guide future research in this emerging field.

**Figure 1:**
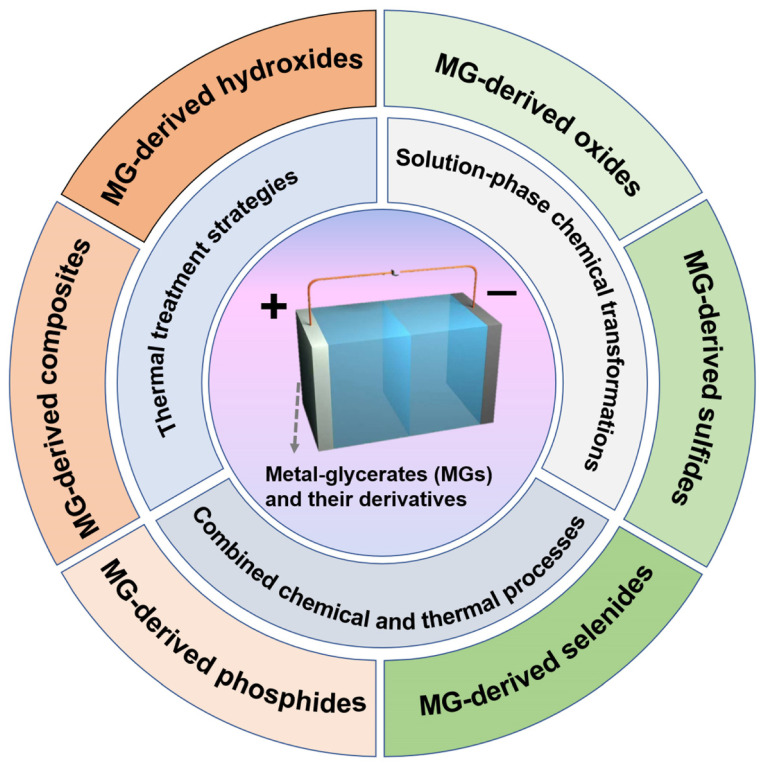
Schematic diagram outlining the strategy for employing metal glycerates and their derivatives in supercapacitor applications.

In this review, we provide a comprehensive summary of recent advances in MGs and their derivatives as electrode materials for SCs, with particular interest in synthetic methods, and especially SC applications. We begin by outlining the synthesis strategies for MGs and their derivatives, followed by an in-depth discussion of their application as high-performance electrodes in SCs (Figure 1). Finally, we outline the current challenges and provide future perspectives on the rational design of MG-based materials for next-generation supercapacitor technologies. This review aims to offer valuable insights and guidance for advancing the development of MG-based materials and accelerating their practical deployment in high-performance SC systems.

## 2. Synthetic Strategies of MGs and Their Derivatives

### 2.1. Synthetic Strategies of MGs

Solvothermal synthesis is a widely employed and effective method for preparing MGs [55,65]. Under solvothermal conditions, multidentate glycerol ligands chelate metal cations to form MG frameworks that, aided by viscosity and hydrogen bonding of glycerol, self-assemble into low-surface-energy spherical aggregates, giving rise to monodisperse microspheres [54,57,64,78]. The type and concentration of metal precursors, surfactants, reaction parameters such as temperature and duration play pivotal roles in determining the morphological and structural characteristics of the resulting MGs [79,80,81]. By precisely adjusting the molar ratios of metal ions, surfactants, and reaction conditions, the nucleation and crystal growth processes can be carefully controlled. Numerous studies have reported the use of various metal salts as metal sources and glycerol as an organic linker in the solvothermal synthesis of MGs, enabling the tailored design of their composition and structure.

The selection of metal precursors allows for the solvothermal synthesis of MGs with a wide range of metal compositions, encompassing single-metal MGs (e.g., Co-glycerate [82], Mn- glycerate [83], Mo-glycerate [84], Fe-glycerate [76]), bimetallic MGs (e.g., NiCo-glycerate [56], CoMn-glycerate [85], CoMo-glycerate [86], NiMn-glycerate [87]), trimetallic MGs (e.g., NiCoMn-glycerate [88], NiCoMo-glycerate [68], ZnMnCo-glycerate [89], NiCoZn-glycerate [72]), and even high-entropy MGs incorporating multiple metal elements [90,91].

**Figure 2:**
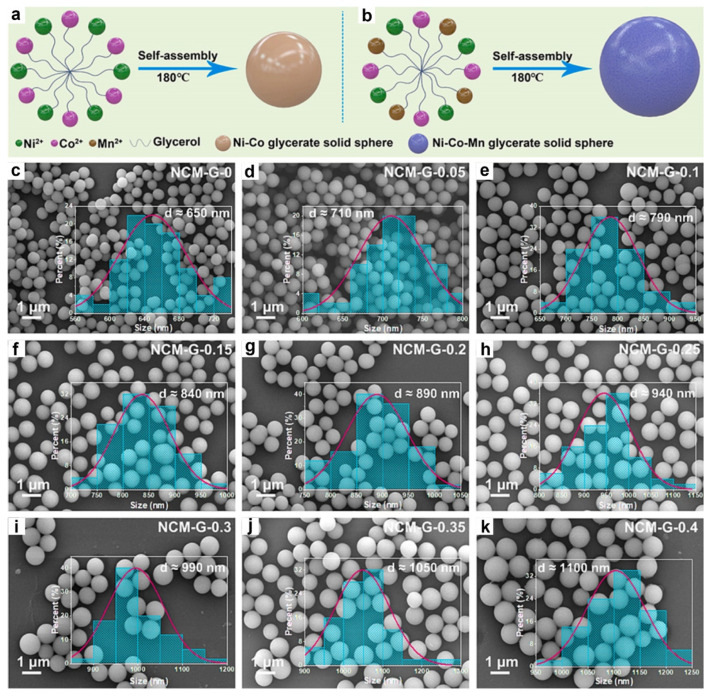
Schematics of sphere formation for (**a**) Ni-Co glycerate and (**b**) Ni-Co-Mn glycerate. FESEM images and size histograms for products prepared with Mn(NO_3_)_2_ at (**c**) 0, (**d**) 0.05, (**e**) 0.10, (**f**) 0.15, (**g**) 0.20, (**h**) 0.25, (**i**) 0.30, (**j**) 0.35, and (**k**) 0.40 mmol. Reproduced with permission [50]. Copyright 2023, Elsevier.

Beyond compositional diversity, the concentration of metal precursors has been shown to significantly influence the particle size of MGs. For example, our group successfully synthesized monodisperse NiCoMn-glycerate solid spheres with tunable diameters ranging from 650 to 1100 nm by varying the amount of Mn(NO_3_)_2_, without employing any surfactants [50]. As illustrated in Figure 2a,c, the approach involved a solvothermal process using Co(NO_3_)_2_, Ni(NO_3_)_2_, and glycerol in isopropanol to produce uniform NiCo-glycerate spheres. Remarkably, upon introducing Mn(NO_3_)_2_ into the reaction system, the spherical morphology was preserved, yet the particle size noticeably increased (Figure 2b,d–k). To gain further insight, we investigated Co-glycerate spheres synthesized at varying concentrations of Co(NO_3_)_2_. The results revealed that higher Co^2+^ concentrations led to larger particle sizes, suggesting that an increased metal ion content enhances collision frequency between metal ions and glycerate molecules, thereby accelerating crystal growth and resulting in larger MG spheres.

**Figure 3:**
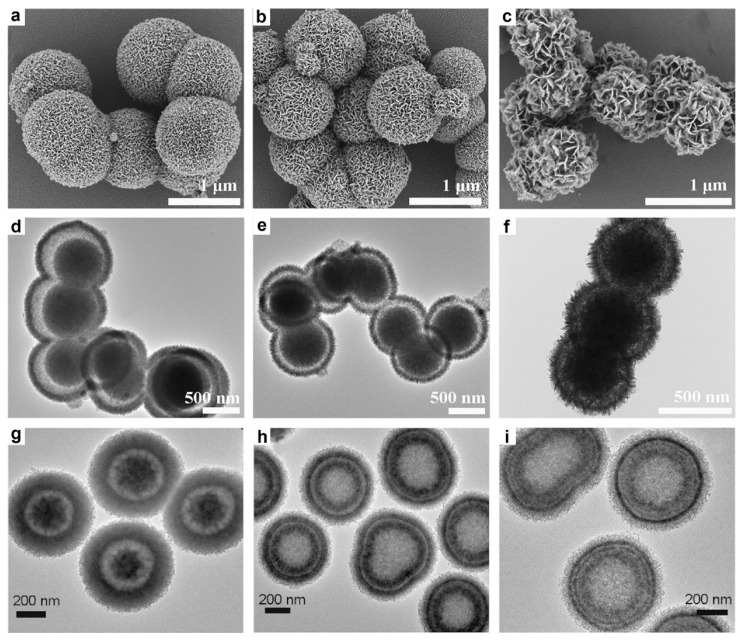
SEM images of (**a**) Co_1_Fe_3_-glycerates, (**b**) Co_1_Fe_1_-glycerates, and (**c**) Co_1_Fe_1_-glycerates; TEM images of (**d**) Co_1_Fe_3_-glycerates, (**e**) Co_1_Fe_1_-glycerates and (**f**) Co_1_Fe_1_-glycerates. Reproduced with permission [92]. Copyright 2025, Elsevier. TEM images of time-evolved precursors collected at (**g**) 3 h, (**h**) 6 h, and (**i**) 8 h. Reproduced with permission [80]. Copyright 2019, Elsevier.

In another study, Kong et al. successfully synthesized a series of CoFe-glycerates featuring yolk-shell microsphere architectures assembled from ultrathin nanosheets, by precisely adjusting the molar ratios of cobalt nitrate and ferric nitrate precursors while maintaining the total metal precursor amount [92]. As shown in Figure 3a–f, the morphology of these CoFe-glycerates varied significantly with the Co/Fe ratio. Co_1_Fe_3_-glycerate formed larger spheres (~1.1 μm) with a dense, three-dimensional flower-like structure composed of vertically aligned nanosheets. As the cobalt content increased, Co_1_Fe_1_-glycerate exhibited smaller spheres (~900 nm) with larger nanosheets and more noticeable gaps, whereas Co_3_Fe_1_-glycerate yielded even smaller spheres (~700 nm) characterized by loosely arranged nanosheets. In addition, Li and co-workers reported the synthesis of three distinct CoCu-glycerate structures by varying the duration of the solvothermal reaction [80]. Remarkably, the number of shells in the resulting materials could be precisely controlled by adjusting the reaction time. As illustrated in Figure 3g–i, single-, double-, and triple-shelled CoCu-glycerates were obtained after reaction times of 3, 6, and 8 h, respectively.

**Figure 4:**
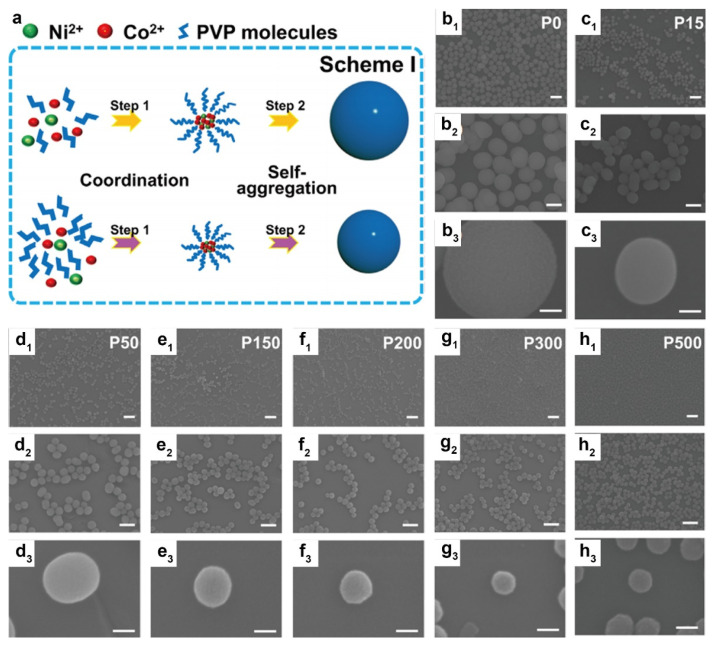
(**a**) Schematic of the PVP-assisted, size-controllable synthesis of NiCo-glycerates. (**b_1_**–**h_3_**) SEM images of NiCo-glycerates obtained with varying PVP mass; scale bars: (**b_1_**–**h_1_**) 1 μm, (**b_2_**–**h_2_**) 500 nm, and (**b_3_**–**h_3_**) 100 nm. Reproduced with permission [81]. Copyright 2020, Wiley-VCH.

Furthermore, the incorporation of surfactants during the synthesis of MGs plays a crucial role in controlling particle size. For instance, Zhang et al. synthesized a series of NiCo-glycerates via a polyvinylpyrrolidone (PVP)-assisted solvothermal method, employing nickel nitrate, cobalt nitrate, and glycerol in an isopropanol solution to produce highly uniform and monodisperse solid nanospheres (Figure 4a) [81]. The particle size of the NiCo-glycerates was precisely tuned by varying the amount of PVP added during synthesis, with quantities ranging from 0 to 500 mg resulting in precursor diameters decreasing from 550 nm to 97 nm. As shown in Figure 4(b_1_)–(h_3_), all samples exhibited consistent spherical morphologies and smooth surfaces across different sizes, demonstrating good uniformity and dispersion.

### 2.2. Synthesis Strategies of Metal-Glycerate Derivatives

Beyond the precise design of MG precursors, the strategies employed for their conversion into MG-derived materials are crucial for precisely tailoring the nanostructure and composition of the resulting functional materials. Due to their relatively weak coordination environments, MGs possess high chemical reactivity, enabling controlled structural transformations during subsequent processing [54]. This inherent reactivity makes MGs versatile precursors for synthesizing a wide range of nanostructured materials, including various metal compounds and composites [64]. Nevertheless, the intrinsic chemical and thermal instability of MGs necessitate careful selection of conversion methods to ensure the preservation of desired morphological and structural features [93]. Broadly, the synthesis routes for MG-derived materials can be classified into three primary categories: (1) thermal treatment strategies; (2) solution-phase chemical transformations; and (3) combined chemical and thermal processes.

#### 2.2.1. Thermal Treatment Strategies

Thermal treatment is widely employed for converting MG precursors into their derivatives due to its simplicity and precise control over material properties [94]. This process eliminates moisture and volatile components at elevated temperatures and often induces the formation of hollow nano- and microscale structures, driven by shrinkage and adhesion effects under nonequilibrium conditions [95]. The choice of atmosphere plays a crucial role in determining the final product composition and morphology: oxidative environments typically yield metal oxides that retain the precursor morphology and develop porous structures, whereas inert atmospheres promote partial carbonization, leading to metal/carbon composites [96,97,98]. Moreover, integrating gas–solid reactions such as sulfidation, phosphidization, and selenization during thermal treatment enables the formation of metal sulfides, phosphides, or selenides with tailored compositions and enhanced electrochemical performance [99,100].

**Figure 5:**
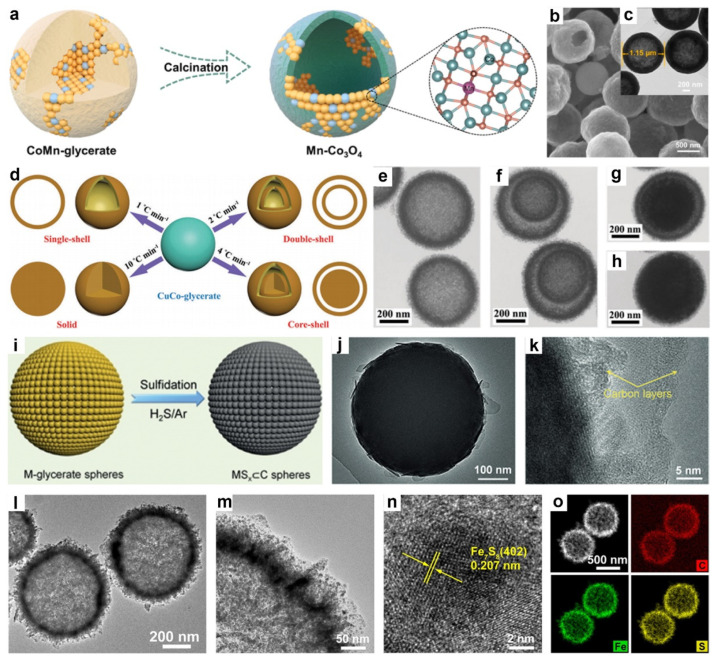
(**a**) Schematic of the sequential route to Mn-Co_3_O_4_, (**b**) SEM and (**c**) TEM images of Mn-Co_3_O_4_. Reproduced with permission [101]. Copyright 2024, Elsevier. (**d**) Synthesis of CuCo_2_O_4_ microspheres with hierarchical internal structures. (**e**–**h**) TEM images of the single-shell, double-shell, core–shell and solid CuCo_2_O_4_ microspheres. Reproduced with permission [102]. Copyright 2017, Royal Society of Chemistry. (**i**) Schematic of the hierarchical MS_x_⊂C sphere formation. (**j**) TEM and (**k**) HRTEM images of V_2_S_3_⊂C spheres. Reproduced with permission [103]. Copyright 2019, Wiley-VCH. (**l**,**m**) TEM images, (**n**) HRTEM image, and (**o**) element mapping images of Fe_7_S_8_@HCSs. Reproduced with permission [97]. Copyright 2022, Elsevier.

In 2024, Xie and co-workers synthesized Mn-doped Co_3_O_4_ (Mn-Co_3_O_4_) with a porous hollow spherical architecture via the calcination of CoMn-glycerate precursors in air (Figure 5a) [101]. As shown in Figure 5b,c, the resulting Mn-Co_3_O_4_ exhibited a uniform single-shelled hollow structure with an average diameter of ~1.15 μm. Similarly, Zhang et al. prepared hollow Co_3_O_4_/CuO nanospheres by calcining hollow CuCo-glycerate precursors in air [94]. They systematically examined the influence of the Co/Cu atomic ratio on the microstructural evolution of both the glycerate precursors and the resulting oxide materials. Notably, the incorporation of copper not only facilitated the transformation from solid Co-glycerate to hollow CuCo-glycerate nanospheres but also significantly enhanced the electrochemical performance of the resulting Co_3_O_4_/CuO composites.

In another study, Kaverlavani and colleagues synthesized CuCo_2_O_4_ microspheres with diverse hollow architectures using a simple and cost-effective self-template method (Figure 5d) [102]. In this process, CuCo-glycerate microspheres were first fabricated via a solvothermal approach, followed by calcination at different heating rates. This method produced CuCo_2_O_4_ microspheres with varied internal structures, including single-shell, double-shell hollow spheres, core–shell spheres, and solid spheres. TEM images demonstrated that the heating rate significantly influenced the resulting morphologies, yielding single-shell (Figure 5e), double-shell (Figure 5f), core–shell (Figure 5g), and solid sphere (Figure 5h) structures.

In addition, Yu’s group developed a general strategy for synthesizing carbon-coated metal sulfide (MS⊂C) spheres by first preparing uniform MG precursors via solvothermal synthesis, followed by thermal sulfidation in an H_2_S/Ar atmosphere (Figure 5i) [103]. For instance, annealing V-glycerate yielded uniform V_2_S_3_⊂C spheres, in which nanoscale V_2_S_3_ crystals were embedded within an in situ formed carbon matrix. Specifically, the V-glycerate spheres initially decompose to form V_2_O_3_ nanocrystals embedded within an in situ generated carbon matrix. Upon further heating, sulfidation occurred through the replacement of O^2−^ ligands by S^2−^, leading to the formation of V_2_S_3_ nanocrystals confined within the carbon matrix. This confinement effectively prevented excessive particle growth, resulting in uniformly dispersed nanoscale V_2_S_3_ embedded in a conductive carbon shell (Figure 5j,k).

Similarly, Xu et al. synthesized ultrafine Fe_7_S_8_ nanocrystals embedded within hollow carbon nanospheres (Fe_7_S_8_@HCSs) through low-temperature sulfurization of hollow Fe-glycerate nanospheres [97]. In the synthesis, hollow Fe-glycerate spheres were first prepared via a solvothermal method, where the amount of deionized water was critical in achieving the hollow structure. Subsequent calcination in an Ar atmosphere converted the glycerate into carbon and transformed Fe^3+^ into Fe_3_O_4_ nanoparticles, forming Fe_3_O_4_@HCSs. Finally, sulfurization at 350 °C in an H_2_/Ar atmosphere with sublimed sulfur powder produced Fe_7_S_8_@HCSs, effectively preventing nanoparticle aggregation typically observed during high-temperature sulfidation. As shown in Figure 5l,m, the resulting Fe_7_S_8_@HCSs exhibited a hollow spherical structure with a diameter of ~670 nm, featuring a nanosheet-assembled shell and uniformly dispersed Fe_7_S_8_ nanoparticles ~10 nm in size within the carbon matrix. HRTEM (Figure 5n) revealed a lattice spacing of 0.207 nm, corresponding to the d_402_ planes of Fe_7_S_8_, while EDX elemental mapping (Figure 5o) confirmed the uniform distribution of C, S, and Fe, with weight ratios of 9.1%, 35.2%, and 55.7%, respectively.

#### 2.2.2. Solution-Phase Chemical Transformations

In addition to thermal treatment methods, solution-phase chemical transformations such as ion exchange offer an effective route for converting MG precursors into nanostructured derivatives with tunable compositions and architectures [85,87]. These solid–liquid reactions often proceed under relatively mild conditions, particularly when the interaction between metal ions and organic ligands in the MG framework is weaker than that between the metal ions and incoming inorganic anions [104,105]. Such transformations can induce significant changes in the morphology and internal structure of the materials, enabling the formation of diverse functional nanostructures. Moreover, solution-phase processes preserve delicate nanostructures and allow precise control over composition and particle size, which is crucial for optimizing electrochemical properties.

**Figure 6:**
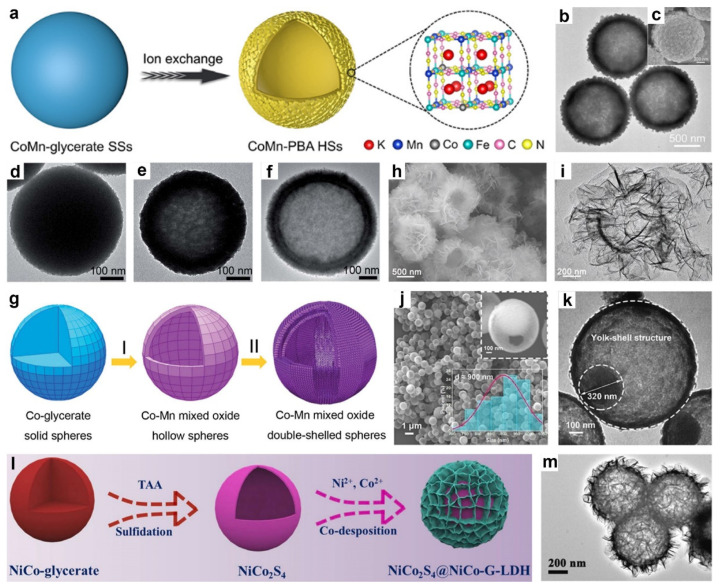
(**a**) The synthetic process of CoMn-PBA HSs. (**b**) TEM image and (**c**) SEM image of CoMn-PBA HSs. Reproduced with permission [106]. Copyright 2019, Wiley-VCH. TEM images of products from the Co-glycerate/KMnO_4_ reaction at (**d**) 0, (**e**) 10, and (**f**) 60 min. (**g**) Schematic illustrating the evolution to Co-Mn mixed-oxide double-shell hollow spheres. Reproduced with permission [107]. Copyright 2019, Royal Society of Chemistry. (**h**) SEM image and (**i**) TEM image of CN-xFe HMs. Reproduced with permission [108]. Copyright 2022, Wiley-VCH. (**j**) FESEM of NCM-S-0.25 with insets showing a broken-structure SEM (top right) and the size distribution profile (bottom right). (**k**) TEM image of NCM-S-0.25. Reproduced with permission [50]. Copyright 2023, Elsevier. (**l**) Schematic of NiCo_2_S_4_@NiCo-G-LDH formation. (**m**) TEM image of NiCo_2_S_4_@NiCo-G-LDH. Reproduced with permission [111]. Copyright 2022, Elsevier.

In 2021, Lou’s group demonstrated a self-templating synthesis of cobalt-substituted Mn-rich Prussian blue analog hollow spheres (CoMn-PBA HSs), in which solvothermally prepared CoMn-glycerate solid spheres were converted into hierarchical hollow architectures through an anion-exchange process, forming a shell composed of interconnected nanocubes (Figure 6a) [106]. The TEM (Figure 6b) and SEM (Figure 6c) images revealed that CoMn-PBA HSs possessed a well-defined hierarchical hollow structure with ~70 nm nanocube subunits uniformly assembled on the surface, forming shells ~200 nm thick and an overall diameter of ~1.2 μm. Moreover, Guo et al. developed a self-templated method to synthesize Co-Mn mixed oxide double-shelled hollow spheres by transforming solid Co-glycerate spheres through redox reactions with KMnO_4_ followed by Ostwald ripening at mild temperatures [107]. The formation mechanism of the Co-Mn mixed oxide double-shelled hollow spheres was elucidated through a time-resolved morphological evolution study (Figure 6d–f). Initially, the Co-glycerate precursors retain a nearly intact spherical shape with slight surface roughening (Figure 6d). After 10 min of hydrothermal treatment, internal voids begin to emerge, leading to the formation of single-shelled hollow structures (Figure 6e). Following a 1 h reaction with KMnO_4_, a well-defined double-shelled morphology is observed, featuring two distinct shells and internal cavities (Figure 6f). The appearance of randomly oriented nanoflakes on the outer shell indicates the formation of MnCo_2_O_4_ via an Ostwald ripening process, wherein smaller nanoparticles dissolve and redeposit to construct the secondary shell. A schematic representation of this formation pathway is shown in Figure 6g.

In one representative study, Zhang and co-workers reported the synthesis of NiCo-LDH hollow spheres through a simple ion-exchange strategy using Co-glycerate spheres as sacrificial templates [105]. The Co-glycerate precursors were first obtained via a solvothermal method, followed by treatment with Ni(NO_3_)_2_ in an ethanol-based solution. During this process, ion exchange occurred, leading to the formation of NiCo-LDH hollow structures. Notably, protons, hydroxyl groups, and Ni^2+^ ions generated from the hydrolysis of Ni(NO_3_)_2_ played dual roles: etching the Co-glycerate core and reacting with released Co ions to facilitate the in situ growth of the NiCo-LDH shell. Similarly, Che’s group synthesized trimetallic CoNiFe carbonate hydroxide hierarchical hollow microflowers (CN-xFe HMs) employing CoNi-glycerate nanospheres as sacrificial templates [108]. Specifically, uniform CoNi-glycerate nanospheres were first prepared via a modified solvothermal method. Upon hydrothermal treatment, partial oxidation of Co^2+^ to Co^3+^ occurred, and the in situ generated OH^−^ and CO_3_^2−^ species reacted with Co^2+^, Co^3+^, and Ni^2+^ to form nanosheet-like carbonate hydroxides on the surface, leading to yolk-shell structures. These intermediates were subsequently immersed in Fe(NO_3_)_3_ solution, enabling cation exchange and partial substitution of Co species by Fe^3+^, ultimately resulting in well-defined hollow architectures. The hierarchical hollow microflower morphology of CN-xFe HMs was confirmed by the SEM (Figure 6h) and TEM (Figure 6i) images. In a more recent example, Jia and co-workers synthesized Ce-doped nickel/cobalt hydroxide hierarchical hollow spheres (NiCoCe-OH) through a two-step strategy [109]. First, NiCo-glycerate solid spheres with smooth surfaces were obtained via a solvothermal method and subsequently converted into hierarchical hollow structures composed of interconnected nanosheets through a hydrolysis reaction, driven by the Kirkendall effect. In the second step, a cation-exchange process was conducted by introducing Ce ions into the NiCo-OH framework, with reaction durations ranging from 1 to 8 h to modulate Ce content and morphological characteristics. Among the resulting materials, NiCoCe-OH-4 (4 represents 4 h) exhibited a well-defined hollow structure, featuring ultrathin, curly nanosheets and a uniform distribution of Ce dopants.

In addition, yolk-shelled amorphous Ni-Co-Mn sulfide spheres were synthesized by our group via a sulfidation treatment of Ni-Co-Mn glycerate solid spheres using thioacetamide (TAA) in ethanol solution [50]. During this process, S^2−^ released from the thermal decomposition of TAA reacted with the metal glycerate precursor. Initially, an anion exchange between S^2−^ and the metal species at the surface led to the formation of a core–shelled Ni-Co-Mn glycerate@Ni-Co-Mn sulfide structure. As the reaction progressed, inward diffusion of S^2−^ combined with a faster outward diffusion of metal ions resulted in the growth of the sulfide shell and the formation of an internal void. Eventually, a secondary sulfide layer formed around the core, giving rise to well-defined yolk-shelled Ni-Co-Mn sulfide spheres upon completion of the transformation. FESEM images (Figure 6j) distinctly demonstrate the yolk-shelled hollow structure of the Ni-Co-Mn sulfide with an average diameter of ~900 nm, while fractured spheres provide further evidence of its hollow structure. TEM image (Figure 6k) further confirms the yolk-shelled architecture, revealing an inner core (yolk) diameter of about 320 nm and an outer shell thickness of approximately 40 nm. Similarly, Yang and colleagues reported the synthesis of Cu_7_Se_4_-Cu_x_Co_1−x_Se_2_ double-shelled hollow nanospheres via a facile self-templated hydrothermal anion exchange method [110]. In this approach, CuCo-glycerate nanospheres act as sacrificial templates and undergo a one-step hydrothermal reaction during which Se^2−^ replace glycerate anions, leading to the in situ formation of well-defined double-shelled hollow architectures. By precisely controlling the reaction time, the morphology and composition of the composite can be optimized without requiring separate synthesis of the individual components.

In another work, Li and co-workers synthesized NiCo_2_S_4_@NiCo-G-LDH hierarchical core–shelled heterostructure via a two-step strategy (Figure 6l) [111]. Firstly, uniform NiCo_2_S_4_ hollow nanospheres were obtained by hydrothermal vulcanization of NiCo-glycerate precursors. Then, ultrathin NiCo-LDH nanosheets were grown on the surface of the hollow NiCo_2_S_4_ nanospheres via a co-deposition process, during which glucose molecules were introduced to serve as interlayer guests. As shown in Figure 6m, the NiCo_2_S_4_@NiCo-G-LDH exhibited a well-defined core–shelled hollow architecture, in which glucose-intercalated NiCo-LDH nanosheets are vertically anchored onto the surface of NiCo_2_S_4_ hollow nanospheres. Similarly, Wang et al. synthesized hierarchical multi-cavity hollow Ni-Co bimetallic sulfide composite (Ni_x_Co_1−x_S_2_/NiCo_2_S_4_) via a multistep approach involving solvothermal coordination, interfacial self-assembly, and in situ sulfurization [33]. Specifically, NiCo-glycerate microspheres were first prepared from Ni^2+^/Co^2+^ ions and glycerol. Upon treatment with 2-methylimidazole, surface metal ions partially dissociated and coordinated with the ligand, forming NiCo-ZIF nanoparticles on the NiCo-glycerate surface, yielding a dual-template structure. Subsequent sulfurization with TAA under solvothermal conditions produced S^2−^ that reacted with metal species to form sulfides. The Kirkendall effect facilitated hollow structure formation, with Ni_x_Co_1−x_S_2_ nanoparticles inside and NiCo_2_S_4_ on the outer shell, resulting in a hierarchical multicavity architecture. This well-defined structure offered abundant active sites and enhanced mechanical integrity, contributing to improved electrochemical performance.

#### 2.2.3. Combined Chemical and Thermal Processes

Combined chemical and thermal strategies synergize solution-phase reactivity with high-temperature treatment, enabling refined control over composition, crystallinity, and hierarchical architecture [76,112]. Typically, these methods involve sequential chemical modification and thermal conversion steps, performed in either order, to tailor complex hollow architectures. In one route, MG precursors undergo chemical transformation such as ion exchange, coordination, or hydrolysis, facilitating the incorporation of hetero-elements or the formation of hollow or yolk-shell intermediates [66,113]. Subsequent thermal treatment (e.g., calcination, phosphorization, sulfurization) induces phase conversion and crystallization, often via the Kirkendall effect, yielding hollow structures with preserved external morphology [100,114]. Alternatively, thermal pretreatment can first generate stable metal oxide frameworks, followed by post-synthetic chemical modifications (e.g., anion exchange or surface functionalization) to produce sulfides, phosphides, or hybrid composites without compromising structural integrity [96,115,116]. Such combined approaches effectively retain delicate architectures during processing and enable the design of multi-shelled, hierarchical, and multicavity systems. The synergistic tuning of composition and architecture enhances electronic conductivity, structural robustness, and active site accessibility, all of which are critical for high-performance SCs.

**Figure 7:**
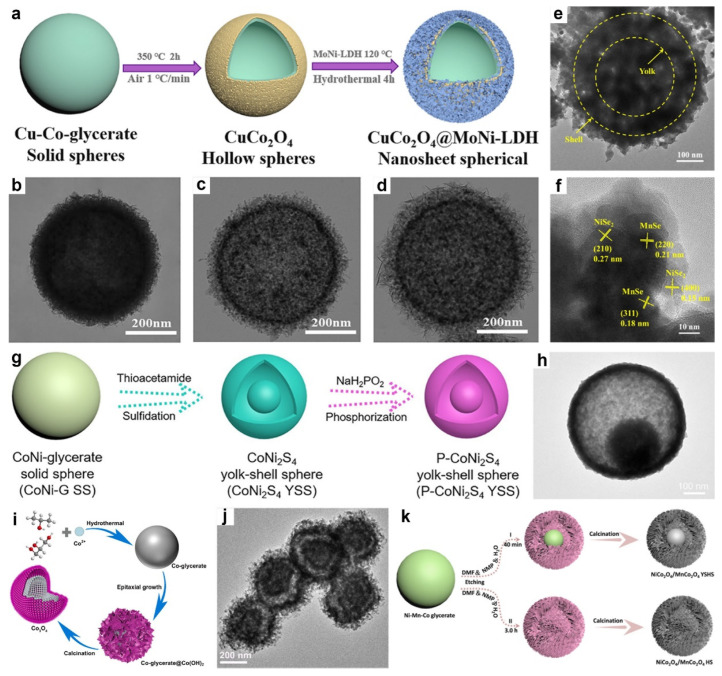
(**a**) Schematic of the synthesis method followed to obtain CuCo_2_O_4_@MoNi-LDH formation and (**b**–**d**) corresponding images at each stage. Reproduced with permission [117]. Copyright 2023, Elsevier. (**e**) TEM image and (**f**) HRTEM image of NiMnSe_3_. Reproduced with permission [118]. Copyright 2022, American Chemical Society. (**g**) Illustration of the preparation of P-CoNi_2_S_4_ YSSs. (**h**) TEM image of P-CoNi_2_S_4_ YSSs. Reproduced with permission [119]. Copyright 2021, Wiley-VCH. (**i**) Schematic illustration of structural evolution of hollow double-shelled Co_3_O_4_ sphere. (**j**) TEM image of hollow double-shelled Co_3_O_4_ sphere. Reproduced with permission [120]. Copyright 2023, Elsevier. (**k**) Schematic representation of a controllable strategy to construct NiCo_2_O_4_/MnCo_2_O_4_ hollow structures. Reproduced with permission [88]. Copyright 2023, Elsevier.

In 2023, Zhang et al. proposed a stepwise synthesis strategy for constructing CuCo_2_O_4_@MoNi-LDH core–shelled hollow nanostructures via sequential thermal calcination and hydrothermal treatment (Figure 7a) [117]. Specifically, the solvothermally prepared CuCo-glycerate solid spheres (Figure 7b) were first annealed at elevated temperatures, leading to the formation of hollow CuCo_2_O_4_ microspheres with roughened surfaces (Figure 7c). The shell exhibited evenly distributed crystalline grains, indicating a uniform structural transformation. Subsequently, MoNi-LDH nanosheets were grown on the surface through a one-step hydrothermal process. As shown in Figure 7d, the nanosheets were conformally coated, forming a well-defined core–shelled hollow architecture. In a related study, Xu and co-workers developed a multistep synthesis strategy to fabricate yolk-shelled hollow NiMnSe_3_ nanospheres by integrating solvothermal treatment, thermal oxidation, and hydrothermal selenification [118]. Initially, Ni-Mn glycerate precursors were prepared via solvothermal synthesis using glycerol as a key structure-directing agent. Glycerol not only facilitated the formation of uniform spheres but also enabled Ostwald ripening during the reaction, wherein the internal glycerate cores gradually shrank while the outer shell thickened, ultimately giving rise to a distinct yolk-shelled hollow configuration. Subsequent calcination in air converted the organic framework into Ni-Mn oxide microspheres with preserved hollow interiors and enhanced structural rigidity. In the final step, the oxide spheres were exposed to selenide ions under hydrothermal conditions, resulting in the formation of porous NiMnSe_3_. As shown in Figure 7e, the yolk-shelled hollow structures were maintained post-selenization, while nanosheets composed of MnSe and NiSe_2_ (Figure 7f) uniformly emerged on the shell surface, forming a hierarchical composite architecture.

Lou’s group synthesized phosphorized CoNi_2_S_4_ (P-CoNi_2_S_4_) yolk-shelled hollow spheres via a sequential two-step strategy involving hydrothermal sulfidation followed by gas-phase phosphorization (Figure 7g) [119]. Initially, CoNi-glycerate precursors were converted into CoNi_2_S_4_ yolk-shelled hollow structure through anion exchange and chemical etching in a TAA solution under hydrothermal conditions, resulting in microspheres with rough surfaces and well-defined interior voids. In the subsequent phosphorization step, these sulfides were phosphorized via a gas–solid reaction, during which phosphorus atoms were introduced to partially substitute the chalcogen components. As shown in Figure 7h, the resulting P-CoNi_2_S_4_ preserved its yolk-shelled hollow structure with a ~35 nm porous shell composed of ultrafine nanoparticles, effectively promoting ion diffusion and active site accessibility.

In addition, Fu’s group developed a multi-step synthetic route to construct hierarchical double-shelled hollow Co_3_O_4_ nanospheres, integrating solvothermal precursor formation, solution-phase shell growth, and controlled thermal treatment (Figure 7i) [120]. In the first step, Co-glycerate solid spheres were synthesized through a one-step solvothermal method and used as sacrificial templates. Subsequently, the introduction of Co(NO_3_)_2_ and 2-methylimidazole at room temperature induced the in situ growth of Co(OH)_2_ nanosheets on the surface of the Co-glycerate spheres, forming a well-defined Co-glycerate@Co(OH)_2_ core–shelled structure. The composite was then subjected to calcination, during which a sequence of thermally induced structural transformations occurred. Initially, the outer Co(OH)_2_ shell was rapidly oxidized to form a rigid Co_3_O_4_ layer firmly attached to the shrinking core. As heating progressed, the Co-glycerate core underwent oxygenolysis and significant inward contraction, leading to its gradual detachment from the outer shell. This mismatch in contraction behavior, governed by the different thermal responses of the inner and outer layers, ultimately resulted in the formation of hierarchical double-shelled hollow Co_3_O_4_ architecture (Figure 7j).

Moreover, Li et al. developed a two-step strategy to construct Co_9_S_8-x_ core–shelled hollow spheres with tunable sulfur vacancy concentrations, using Co-glycerate as the starting precursor [121]. In the first step, the solvothermally prepared Co-glycerate underwent sulfidation with TAA, during which S^2−^ reacted with cobalt centers to form Co_9_S_8_ featuring a core–shelled hollow structures. These Co_9_S_8_ were subsequently annealed under an Ar/H_2_ atmosphere at 300 °C, 400 °C, or 500 °C, yielding core–shelled hollow Co_9_S_8-x_ spheres with controlled sulfur deficiency, designated as Co_9_S_8-x_-L, Co_9_S_8-x_-M, and Co_9_S_8-x_-H, respectively.

In a related work, hollow and yolk-shelled NiCo_2_O_4_/MnCo_2_O_4_ spheres were synthesized by Shi and co-workers via a template-assisted hydrothermal etching strategy followed by calcination (Figure 7k) [88]. Initially, solid NiMnCo-glycerate spheres were synthesized via a solvothermal route and served as sacrificial templates. These were then subjected to hydrothermal treatment in a mixed solvent system of the water, Nmethylpyrrolidone (NMP), and N, N-dimethylformamide (DMF), where partial dissolution of the core accompanied by nanosheet shell formation induced progressive structural transformation. By precisely adjusting the reaction duration, the morphology evolved from dense spheres to yolk-shelled structures, and eventually to completely hollow architectures. This transition was governed by sequential surface reconfiguration, inward etching-driven core–shell separation, and full template degradation. Final thermal annealing converted the intermediate structures into crystalline NiCo_2_O_4_ and MnCo_2_O_4_, while preserving the hollow or yolk-shelled structures.

## 3. Application of Metal-Glycerates and Their Derivatives in Supercapacitor Electrode Materials

### 3.1. Metal-Glycerate as Electrode Materials for Supercapacitor

MGs, a subclass of metal-alkoxides, have emerged as promising electrode materials for SCs owing to their layered and amorphous structures [54]. Their lamellar architecture provides abundant interlayer spacing, which facilitates efficient ion diffusion and electrolyte access. Concurrently, the amorphous nature introduces abundant grain boundaries and disordered coordination environments, providing a high density of electroactive sites and multiple ion transport channels [78]. Under alkaline conditions, MGs can be in situ transformed into metal oxyhydroxides, the actual redox-active species during charge–discharge processes [122]. This structural adaptability, combined with their flexible framework, contributes to enhanced cycling stability.

**Figure 8:**
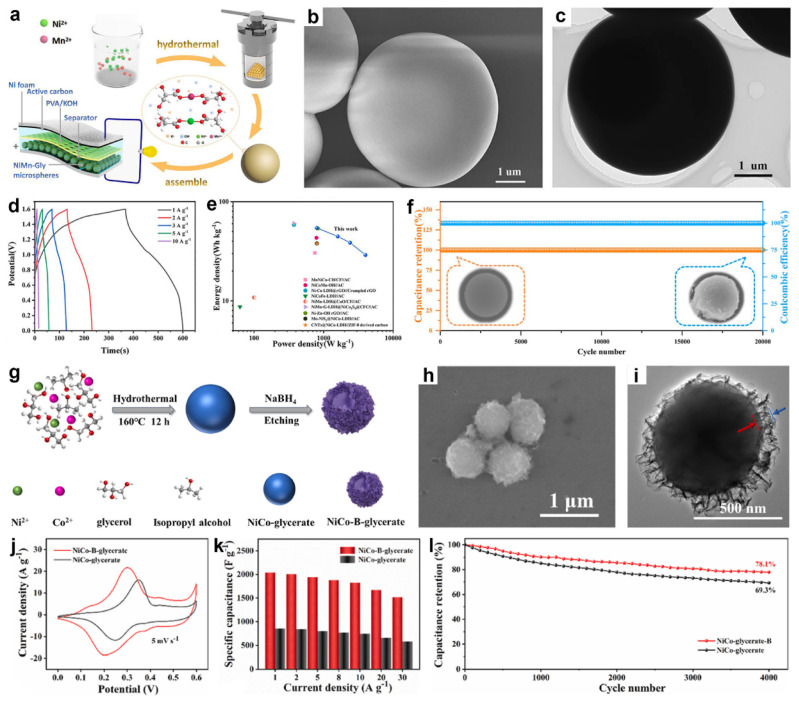
(**a**) Schematic of NiMn-Gly formation and the NiMn-Gly-1//AC solid-state device. (**b**) SEM and (**c**) TEM images of NiMn-Gly-1. (**d**) GCD curves at different current densities, (**e**) Ragone plots, and (**f**) cycling stability of NiMn-Gly-1//AC device. Reproduced with permission [78]. Copyright 2022, Elsevier. (**g**) Schematic of the formation process of NiCo-B-glycerate. (**h**) SEM and (**i**) TEM images of NiCo-B-glycerate. (**j**) The CV curves at 5 mV s^−1^ of NiCo-B-glycerate. (**k**) Specific capacities and (**l**) cycling stability of NiCo-B-glycerate and NiCo-glycerate. Reproduced with permission [55]. Copyright 2024, Elsevier.

**Figure 9:**
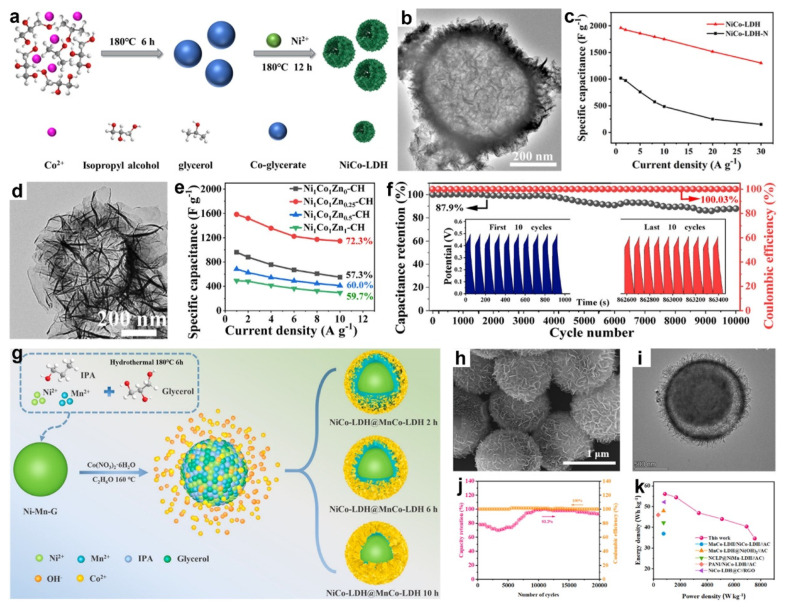
(**a**) Schematic of NiCo-LDH hollow sphere fabrication routes. (**b**) TEM image of NiCo-LDH hollow sphere. (**c**) Specific capacities at different current densities. Reproduced with permission [105]. Copyright 2022, Elsevier. (**d**) TEM image of NiCoZn-CH. (**e**) Specific capacitance of the electrodes plotted against current density. (**f**) Cycling stability of Ni_1_Co_1_Zn_0.25_-CH electrode. Reproduced with permission [72]. Copyright 2023, Royal Society of Chemistry. (**g**) Schematic diagram of the synthesis of NiCo-LDH@MnCo-LDH. (**h**) SEM and (**i**) TEM images of NiCo-LDH@MnCo-LDH. (**j**) Cycling stability and coulombic efficiency, and (**k**) Ragone plot of NiCo-LDH@MnCo-LDH//AC ASC. Reproduced with permission [87]. Copyright 2025, Elsevier.

Several studies have demonstrated the feasibility of using pristine metal glycerates directly as SCs electrode materials. For example, Ding et al. synthesized uniform NiMn-glycerate (NiMn-Gly) microspheres via a solvothermal coordination reaction between glycerate ligands and Ni^2+^/Mn^2+^, which were subsequently used to assemble an all-solid-state NiMn-Gly//activated carbon (AC) SC device with a PVA/KOH gel electrolyte (Figure 8a) [78]. SEM image (Figure 8b) revealed that the microspheres possess a uniform spherical morphology with diameters of 3–6 μm and smooth surfaces. TEM image (Figure 8c) further confirmed their solid internal structure with well-defined, nonporous boundaries. As shown in Figure 8d, the all-solid-state device demonstrated stable galvanostatic charge–discharge (GCD) behavior within a voltage window of 0–1.6 V at varying current densities, delivering specific capacitances of 244.9, 201.0, 173.7, 130.5, and 72.0 C g^−1^ at 1, 2, 3, 5, and 10 A g^−1^, respectively. The nearly negligible IR drop observed in the GCD curves reflects low internal resistance and efficient charge transport. Notably, the device achieved a maximum energy density of 54.4 Wh kg^−1^ at a power density of 800 W kg^−1^ (Figure 9e). As shown in Figure 9f, the device retained nearly 100% capacitance and coulombic efficiency after 20,000 cycles at 10 A g^−1^, maintaining intact microsphere morphology and good structural stability.

In another study, Zhang et al. developed boron-doped NiCo-glycerate spheres (NiCo-B-glycerate) with a distinctive sphere@nanosheet structure via a two-step strategy (Figure 8g) [55]. Initially, uniform NiCo-glycerate solid spheres were synthesized through a solvothermal method. A subsequent NaBH_4_ treatment served dual roles, acting both as a boron source and a chemical etchant, simultaneously incorporating B atoms into the framework and inducing surface reconstruction. This process led to the formation of wrinkled nanosheets on the particle surface, as confirmed by FESEM (Figure 8h) and TEM (Figure 8i) images. The transformation was attributed to the oxygen-scavenging effect of hydride ions (H^−^), which generated surface defects and promote local structural rearrangement. The resulting architecture shortened ion diffusion pathways and improved mechanical adaptability during cycling. As a result, the electrochemical performance was significantly improved. As shown in Figure 8j, both NiCo-glycerate and NiCo-B-glycerate exhibited distinct redox peaks in their CV curves at 5 mV s^−1^, indicative of typical pseudocapacitive behavior. Notably, NiCo-B-glycerate displayed smaller redox peak separation, indicating reduced polarization and faster electron transfer kinetics. Furthermore, NiCo-B-glycerate delivered a markedly higher specific capacitance of 2036 F g^−1^ at 1 A g^−1^, significantly surpassing the 856 F g^−1^ recorded for the undoped NiCo-glycerate (Figure 8k). At a high current density of 30 A g^−1^, it retained 74.6% of its initial capacitance, compared to 68.2% retention for the undoped sample. Durability tests at 10 A g^−1^ further demonstrated superior stability of NiCo-B-glycerate, retaining 78.1% of its initial capacity after 4000 cycles versus 69.3% for the undoped counterpart (Figure 8l). An asymmetric supercapacitor (ASC) constructed with NiCo-B-glycerate achieved an energy density of 58.3 Wh kg^−1^ at 0.8 kW kg^−1^. The superior electrochemical performance of NiCo-B-glycerate was mainly attributed to B doping, which generated a defective coordination environment in NiCo-glycerate and modulated its electronic structure, together with the 2D nanosheet morphology that provided additional accessible active sites and shortened charge-transport pathways.

Overall, pristine MGs as electrodes demonstrate that even without further conversion, their lamellar-amorphous frameworks can deliver competitive capacitance and durability compared with many conventional hydroxides and oxides. The comparison between NiMn-Gly and NiCo-/NiCo-B-glycerates indicates that introducing multimetal synergy, defect engineering (e.g., B doping), and controlled surface reconstruction is crucial for simultaneously enhancing redox kinetics and structural robustness. At the same time, the relatively modest rate capability of pristine MGs highlights their intrinsic constraints, motivating subsequent transformation into derivatives with higher electronic conductivity and richer redox chemistry.

### 3.2. Metal-Glycerate Derivatives as Electrode Materials for Supercapacitor

Recent advancements in metal-glycerate (MG)-derived materials have demonstrated their great promise as electrode candidates for supercapacitors, primarily owing to their tunable morphology, diverse composition, and favorable redox properties [94,123]. Upon thermal or chemical conversion, MG precursors can be transformed into a variety of high-performance electroactive materials such as hydroxides metal oxides, sulfides, phosphides, and selenides, all while retaining or enhancing their original hierarchical architectures [54,124,125]. These derivatives exhibit multiple electrochemical advantages, including abundant electroactive sites, shortened ion/electron transport paths, and improved structural stability during long-term cycling [111,126,127].

#### 3.2.1. MG-Derived Hydroxides

Layered double hydroxides (LDHs), a class of anionic layered functional materials, have attracted attention for use in SCs because of their superior anion-exchange ability, rich redox-active sites, and favorable ion-intercalation characteristics [38,128,129,130]. Building upon these merits, MG-derived LDHs have emerged as particularly promising electrode materials for SCs [87,124]. The incorporation of MGs as precursors offers a controllable route to tailor the composition and morphology of LDHs, thereby enhancing their electrochemical activity. During the conversion process, the in situ generation of hollow or/and porous nanosheet architectures effectively increases the accessible surface area and facilitates rapid ion diffusion [105]. Furthermore, the synergistic effects between multiple metal cations (e.g., Ni, Co, Zn or Mn) in the LDH framework can significantly improve the intrinsic redox kinetics and electrical conductivity [72,122]. As a result, MG-derived LDHs often deliver high specific capacitance, enhanced rate capability, and robust cycling durability, making them attractive candidates for high-performance SCs.

In 2022, Zhang and co-workers synthesized the NiCo-LDH hollow spheres via a facile ion-exchange method, using Co-glycerate as both the cobalt source and sacrificial template (Figure 9a) [105]. The TEM image (Figure 9b) showed that the NiCo-LDH hollow sphere possess an internal cavity surrounded by outer nanosheet shells approximately 50 nm thick. This nanosheet-assembled hollow architecture suppressed nanosheet agglomeration, enlarged the electrolyte-accessible surface area, and shortened ion-diffusion paths by facilitating electrolyte penetration into the interior cavity. Benefiting from these compositional and structural features, the NiCo-LDH hollow spheres delivered a high specific capacitance of 1962 F g^−1^ at 1 A g^−1^ with 66.4% capacitance retention at 30 A g^−1^ (Figure 9c). The NiCo-LDH//AC ASC further achieved an energy density of 62.9 Wh k g^−1^ at 0.8 kW k g^−1^. Thus, with the merits of composition and hollow structure, in a subsequent study, Cheng et al. synthesized NiMn-glycerate solid spheres as sacrificial templates and selectively etched them in a 1-methyl-2-pyrrolidone/water mixture to yield hierarchical Ni-Mn hydroxide hollow spheres [122]. Employed as electrode material for SCs, the Ni-Mn hydroxide delivered a high specific capacitance of 1680 F·g^−1^ at 2.0 A·g^−1^ and 1068 F·g^−1^ at 15 A·g^−1^, while retaining 96.6% of their capacitance after 5500 cycles at 10 A·g^−1^. A Ni-Mn hydroxide//AC ASC reached 42.8 Wh kg^−1^ at 1703 W kg^−1^. The superior electrochemical performance stemmed from the combined advantages of a mesoporous, high surface-area architecture and ultrathin nanosheet subunits that expose abundant active sites and shorten ion/electron diffusion paths; the hollow morphology accommodated volume changes during cycling, enhancing structural stability; and the amorphous, layered character of the nanosheets further facilitated rapid ion/electron transport.

In addition, Liu et al. developed novel flower-like NiCoZn-carbonate hydroxide (NiCoZn-CH) hollow nanospheres through a two-step solvothermal process [72]. As shown in Figure 9d, the resulting Ni_1_Co_1_Zn_0.25_-CH exhibited a flower-like hollow nanospheres with an overall diameter of approximately 800 nm and an internal cavity of about 400 nm. This unique flower-like architecture provided a large electrode-electrolyte contact area, abundant active sites, and shortened ion transport pathways, while the hollow interior effectively mitigated structural stress, enhancing cycling stability. Moreover, the incorporation of Zn^2+^ ions facilitated ion transfer and boosted electrochemical activity. As a result, the Ni_1_Co_1_Zn_0.25_-CH electrode delivered high specific capacitances of 1585.2, 1520.6, 1356.8, 1223.4, 1173.6, and 1145.6 F g^−1^ from 1 to 10 A g^−1^, respectively, retaining ~72.3% of its capacitance between 1 and 10 A g^−1^ (Figure 9e). It also showed outstanding durability and charge efficiency, maintaining 87.9% of its initial capacitance (from 962.8 to 846.5 F g^−1^) with a coulombic efficiency of ~100% after 10,000 cycles at 10 A g^−1^ (Figure 9f). The Ni_1_Co_1_Zn_0.25_-CH//AC ASC delivered 33.7 Wh kg^−1^ at 400 W kg^−1^ and showed 99.9% capacity retention after 15,000 cycles at 10 A g^−1^.

Recently, Zhang et al. reported the synthesis of NiCo-LDH@MnCo-LDH yolk-shell heterostructures via a simple two-step hydrothermal method [87]. As shown in Figure 9g, Ni-Mn glycerate (Ni-Mn-G) spheres were first prepared via a solvothermal reaction of Ni^2+^ and Mn^2+^ in glycerol/isopropanol. Subsequent hydrothermal treatment with cobalt nitrate in ethanol partially etched the spheres through Co^2+^ hydrolysis, releasing Ni^2+^ and Mn^2+^ that coprecipitated with Co^2+^. The combination of slower Co^2+^ and OH^−^ diffusion into the sphere interior and proton-induced etching promoted the growth of NiCo-LDH and MnCo-LDH nanosheets on the outer surfaces, yielding the NiCo-LDH@MnCo-LDH composites. As shown in Figure 9h,i, the resulting material exhibited a yolk-shell nanosheet-sphere architecture. An ASC using NiCo-LDH@MnCo-LDH as the positive electrode and AC as the negative electrode retained 93.2% of its capacitance after 20,000 cycles at 6 A g^−1^ (Figure 9j), and delivered a power density of 831.2 W kg^−1^, an energy density of 56.1 Wh kg^−1^ (Figure 9k).

#### 3.2.2. MG-Derived Oxides

Beyond hydroxides, MG precursors can be transformed into a variety of metal oxides, which are widely explored as electrode materials for SCs due to their high theoretical capacitance, chemical stability, and tunable nanostructures [67,115,123]. MG-derived oxides often retain the hierarchical architectures of the original precursors, such as hollow spheres, nanosheets, or porous frameworks, which provide a large surface area and facilitate rapid ion/electron transport [88,94,107]. Moreover, the uniform distribution of multiple metal cations within the oxide lattice can enhance redox activity and improve electronic conductivity, leading to superior electrochemical performance [68,69,131,132].

**Figure 10:**
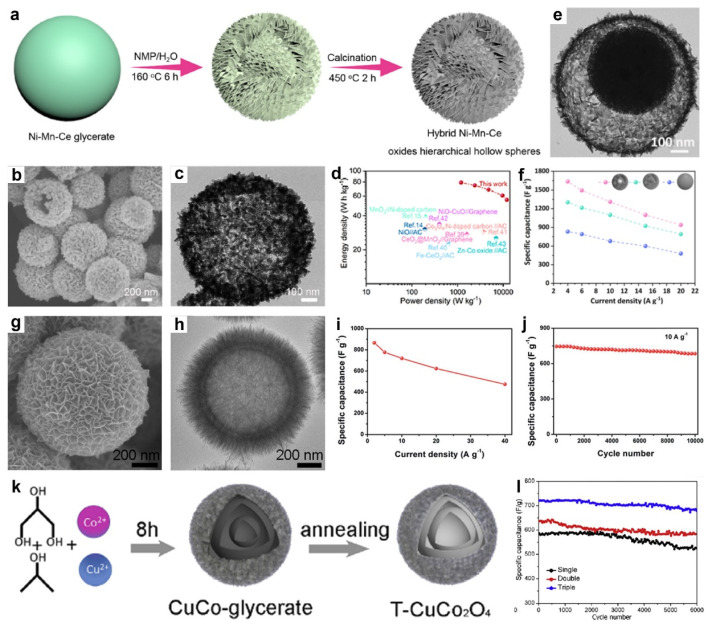
(**a**) Preparation of hybrid Ni-Mn-Ce oxide hierarchical hollow structures. (**b**) SEM and (**c**) TEM images of hierarchical hybrid Ni-Mn-Ce oxide hollow spheres. (**d**) Ragone plot of the solid-state asymmetric device. Reproduced with permission [69]. Copyright 2025, American Chemical Society. (**e**) TEM image of NiCo_2_O_4_/MnCo_2_O_4_ YSHS. (**f**) Specific capacitance of different NiCo_2_O_4_/MnCo_2_O_4_ structures. Reproduced with permission [88]. Copyright 2023, Elsevier. (**g**) FESEM and (**h**) TEM images of Co-Mn mixed oxide double-shelled hollow spheres. (**i**) Specific capacitance as a function of current density and (**j**) cycling performance of Co-Mn mixed oxide double-shelled hollow spheres. Reproduced with permission [107]. Copyright 2023, Royal Society of Chemistry. (**k**) The formation process of T-CuCo_2_O_4_ spheres. (**l**) Cycling stability of different CuCo_2_O_4_. Reproduced with permission [80]. Copyright 2019, Elsevier.

Recently, Zhang et al. reported a simple template strategy to synthesize hybrid Ni-Mn-Ce oxide hierarchical hollow architectures, in which Ni-Mn-Ce glycerate solid spheres were first prepared via a one-pot solvothermal method, then transformed into hierarchical hollow intermediates through treatment in a N-methylpyrrolidone/H_2_O mixture, and finally converted to the hybrid Ni-Mn-Ce oxide hierarchical hollow architectures by calcination (Figure 10a) [69]. The SEM (Figure 10b) and TEM (Figure 10c) images revealed that the hybrid Ni-Mn-Ce oxide possessed a hollow structure, with clear inner voids and a hierarchical shell. Structurally, the mesoporous texture and high surface area enhanced the electrochemical active sites and facilitated electrolyte diffusion. The inner cavities effectively alleviated volume changes during charge–discharge cycles, while the nanosheet-based hierarchical shell shortened ion/electron transport distances, improving rate performance. Compositionally, the hybrid oxide configuration created abundant heterointerfaces, enhancing structural stability and accelerating ion/electron diffusion. Therefore, a solid-state ASC was constructed using Ni-Mn-Ce oxides, achieving a high energy density of 78.9 Wh kg^−1^ at 1199.5 W kg^−1^ (Figure 10d).

In a related study, Hao’s group synthesized hierarchical yolk-shelled NiCo_2_O_4_ spheres via controlled hydrolysis followed by thermal annealing [133]. The resulting structure, featuring an inner spherical core enveloped by nanosheet shells, provided a high surface area of 169.6 m^2^ g^−1^. The NiCo_2_O_4_ exhibited a high capacitance of 835.7 F g^−1^ at 0.5 A g^−1^ and retained 93% of its capacitance after 10,000 cycles. An HSC integrating the NiCo_2_O_4_ with graphene delivered a high energy density of 34.7 Wh kg^−1^ at 395.0 W kg^−1^. Similarly, Shi and colleagues employed Ni-Mn-Co glycerate templates, followed by etching and calcination, to obtain NiCo_2_O_4_/MnCo_2_O_4_ yolk-shelled hollow spheres (YSHS) [88]. As shown in Figure 10e, the as-synthesized NiCo_2_O_4_/MnCo_2_O_4_ YSHS exhibited the well-defined yolk-shelled hollow structures. Correspondingly, the specific capacitance of the NiCo_2_O_4_/MnCo_2_O_4_ YSHS reached 1636, 1494, 1310, and 1100 F g^−1^ at current densities of 4.0–15 A g^−1^, and retained 940 F g^−1^ at 20 A g^−1^ (Figure 10f), indicating good rate capability. In comparison, the solid- and hollow-sphere counterparts exhibited comparable rate behavior but lower capacitance, underscoring the performance advantage imparted by the yolk-shelled hollow architecture. A solid-state ASC employing NiCo_2_O_4_/MnCo_2_O_4_ YSHS was assembled, achieving an energy density of 62.8 Wh kg^−1^ at 1650.4 W kg^−1^.

In addition, Guo et al. reported a facile self-templated route to Co-Mn mixed oxide double-shelled hollow structures [107]. Specifically, Co-glycerate solid spheres were used as templates, then were converted in KMnO_4_ solution via surface redox reactions followed by low-temperature Ostwald ripening. The SEM image (Figure 10g) showed the Co-Mn mixed oxide present a hierarchical nanosheet-assembled exterior. TEM image (Figure 10h) confirmed a double-shelled hollow structure with a distinct gap between the inner and outer shells. Leveraging these compositional and architectural merits, the Co-Mn mixed oxide electrode delivered 860, 778, 720, 624, and 475 F g^−1^ at 2, 5, 10, 20, and 40 A g^−1^ (Figure 10i), respectively, evidencing high-rate capability. Remarkably, as shown in Figure 10j, the electrode exhibits a high reversible capacity of 684 F g^−1^ after 10,000 cycles at 10 A g^−1^ (8.2% loss).

**Figure 11:**
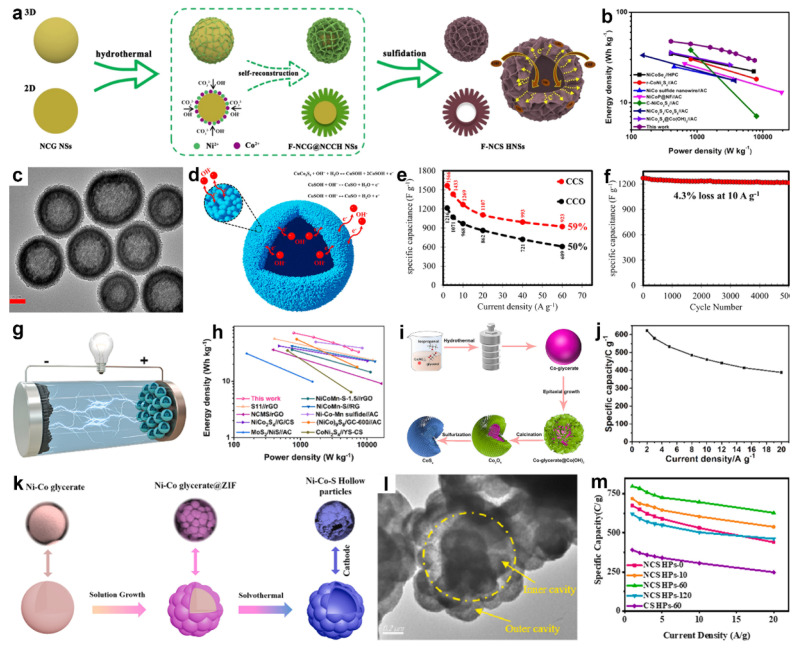
(**a**) Formation mechanism of flower-like NiCo_2_S_4_ hollow nanospheres. (**b**) Ragone diagram of the device. Reproduced with permission [139]. Copyright 2021, American Chemical Society. (**c**) TEM image of CuCo_2_S_4_. (**d**) Schematic of ion transport within the CuCo_2_S_4_ electrode. (**e**) The specify capacitance of the CuCo_2_O_4_ and CuCo_2_S_4_ electrodes at various current densities. (**f**) Cycling stability of CuCo_2_S_4_ electrode. Reproduced with permission [140]. Copyright 2018, American Chemical Society. (**g**) Schematic illustration of the assembled NCM-S-0.25//AC HSC device. (**h**) Ragone plots of the device. Reproduced with permission [50]. Copyright 2023, Elsevier. (**i**) Schematic illustration of the synthesis of hollow double-shell CoS_x_ heterostructures. (**j**) Variation in specific capacity with discharge current density. Reproduced with permission [113]. Copyright 2024, Elsevier. (**k**) Synthesis-route schematic for Ni_x_Co_1−x_S_2_/NiCo_2_S_4_. (**l**) TEM image of Ni_x_Co_1−x_S_2_/NiCo_2_S_4_. (**m**) Specific capacity of the synthesized materials. Reproduced with permission [33]. Copyright 2022, Elsevier.

In another work, Li et al. prepared triple-shelled hollow CuCo_2_O_4_ microspheres (T-CuCo_2_O_4_) by a solvothermal-calcination route (Figure 10k) [80]. The shell number can be precisely tuned by varying the reaction time. The triple-shelled architecture provided abundant electroactive sites and enhanced mechanical robustness, thereby delivering both high specific capacitance and durability. Consequently, T-CuCo_2_O_4_ achieved 691 F g^−1^ at 1 A g^−1^ and 470 F g^−1^ at 20 A g^−1^, whereas the double- and single-shelled counterparts (D-CuCo_2_O_4_ and S-CuCo_2_O_4_) reached 580 and 524 F g^−1^ at 1 A g^−1^, respectively. Moreover, T-CuCo_2_O_4_ retained 68.1% of its specific capacitance upon a tenfold increase in current density, surpassing D-CuCo_2_O_4_ (65.5%) and S-CuCo_2_O_4_ (57.3%), which was attributed to more favorable ion-diffusion kinetics in the hollow framework. As shown in Figure 11l, its specific capacitance decreased only slightly from 702 to 653 F g^−1^ after 6000 cycles (∼7.0% loss), notably outperforming S-CuCo_2_O_4_ (12%) and D-CuCo_2_O_4_ (9.3%).

#### 3.2.3. MG-Derived Sulfides

Transition metal sulfides derived from MG precursors have recently attracted significant attention as promising electrode materials for SCs owing to their high electrical conductivity, rich redox activity, and structural tunability [53,134,135,136]. Typically, MG precursors are sulfurized via thermal or hydrothermal/solvothermal treatment in sulfur-containing media [137,138]. This transformation enhances intrinsic redox kinetics, charge-transfer and ion diffusion efficiency, thereby delivering superior electrochemical performance.

In 2021, Li and co-workers rationally engineered flower-like NiCo_2_S_4_ hollow nanospheres (F-NCS HNS) by coupling surface self-reconstruction with subsequent sulfidation in hydrothermal media [139]. As shown in Figure 11a, uniform NiCo-glycerate nanospheres were first prepared as self-sacrificing templates. During hydrothermal treatment, surface metal ions dissolved and recrystallized into flower-like nanosheet architectures, which were then converted to F-NCS HNS via a facile sulfidation step. Owing to this unique architecture, the F-NCS HNS offered an enlarged surface area with abundant active sites and a porous framework that facilitated ion diffusion, thereby enhancing its electrochemical performance. Consequently, an HSC (F-NCS HNS//AC) delivered an energy density of 47.7 W h kg^−1^ at a power density of 399.9 W h kg^−1^ (Figure 11b).

In another work, Mohammadi et al. synthesized nanoporous CuCo_2_S_4_ hollow spheres by first preparing CuCo-glycerate microspheres through a solvothermal reaction of Cu(NO_3_)_2_ and Co(NO_3_)_2_ in isopropanol/glycerol, followed by sulfurization with thioacetamide in ethanol under solvothermal conditions [140]. TEM image (Figure 11c) distinctly showed a hollow interior surrounded by a porous shell, where the dark edges and lighter centers indicate the cavity structure. Nanoporous CuCo_2_S_4_ hollow spheres excel due to thiospinel conductivity and multi-redox chemistry, hollow microspheres that shorten ion paths and enhance stability, porous shells providing abundant active sites and higher utilization, and nanoscale shell thickness that accelerates electron transport and overall reaction kinetics (Figure 11d). As a result, the CuCo_2_S_4_ electrode delivered higher specific capacitance than CuCo_2_O_4_ (1566 vs. 1216 F g^−1^ at 2 A g^−1^) and superior rate performance compared with CuCo_2_O_4_ (Figure 11e). It also exhibited good durability, with only 4.3% capacitance loss after 5000 cycles at 10 A g^−1^ (Figure 11f). In addition, a CuCo_2_S_4_//AC ASC attained maximum power and energy densities of 16 and 43.65 W h kg^−1^, respectively.

In 2023, our group developed a surfactant-free two-step solvothermal strategy to synthesize size-controllable yolk-shell amorphous Ni-Co-Mn sulfide spheres by first forming Ni-Co-Mn glycerate spheres and subsequently converting them via solvothermal sulfidation [50]. Leveraging trimetallic synergy and the amorphous yolk-shell architecture, the assembled hybrid supercapacitor (NCM-S-0.25//AC, Figure 11g) delivered a high energy density of 71.86 Wh kg^−1^ at 793.56 W kg^−1^ (Figure 11h). In a related study, Chen et al. synthesized double-shelled hollow CoS_x_ spheres employing Co-glycerate templates via a controlled sequence of epitaxial growth, thermal treatment, and sulfuration, with shell formation governed by the Kirkendall effect (Figure 11i) [113]. The hollow double shell architecture afforded robust structural stability, ample electroactive sites, and accelerated electron transport. Therefore, the CoS_x_ electrode delivered specific capacities of 622 and 461 C g^−1^ at 2 and 10 A g^−1^, respectively, retaining 74.1% capacity upon a tenfold increase in current density (Figure 11j). Although the capacity gradually decreased during cycling, 74% of the initial value was still maintained after 10,000 cycles. The assembled CoS_x_//AC HSC achieved an energy density of 38.2 Wh kg^−1^ at a power density of 794.7 W kg^−1^.

In addition, Wang and co-workers synthesized hierarchical multicavity hollow Ni-Co bimetallic sulfide composites using a dual-template-assisted solvothermal approach [33]. As shown in Figure 11k, NiCo-glycerate microspheres were first prepared solvothermally, then dispersed in aqueous 2-methylimidazole to induce surface dissolution and in situ growth of NiCo-ZIF particles, followed by sulfidation with thioacetamide in ethanol under solvothermal conditions, where S^2−^ reacted with Ni/Co species and an ion-diffusion imbalance derived outward deposition to yield hollow multicavity Ni-Co sulfide spheres. Figure 11l confirmed a multicavity hollow architecture in NCS HPs-60, with outer chambers connected to an inner hollow shell and hemispherical subunits (~150 nm radius). This carefully engineered structure maximizes active-site utilization, shortens charge-transport pathways, and mitigates structural collapse during cycling, yielding a high specific capacity of 797 C g^−1^ at 1 A g^−1^ and outstanding rate retention of 78.8% at 20 A g^−1^ (Figure 11m).

#### 3.2.4. MG-Derived Selenides

Compared with oxides, hydroxides, and sulfides, metal selenides derived from MG precursors have recently emerged as highly promising electrode materials for supercapacitors owing to their superior electrical conductivity, higher intrinsic redox activity, and lower bandgap energy [141,142,143]. The substitution of oxygen with selenium effectively enhances the charge-transfer rate and improves reaction reversibility, leading to faster redox kinetics and enhanced capacitance performance [144,145]. Meanwhile, the MG-derived route provides precise control over composition, morphology, and hollow architecture, which helps maintain mechanical integrity and ensures stable long-term cycling.

**Figure 12:**
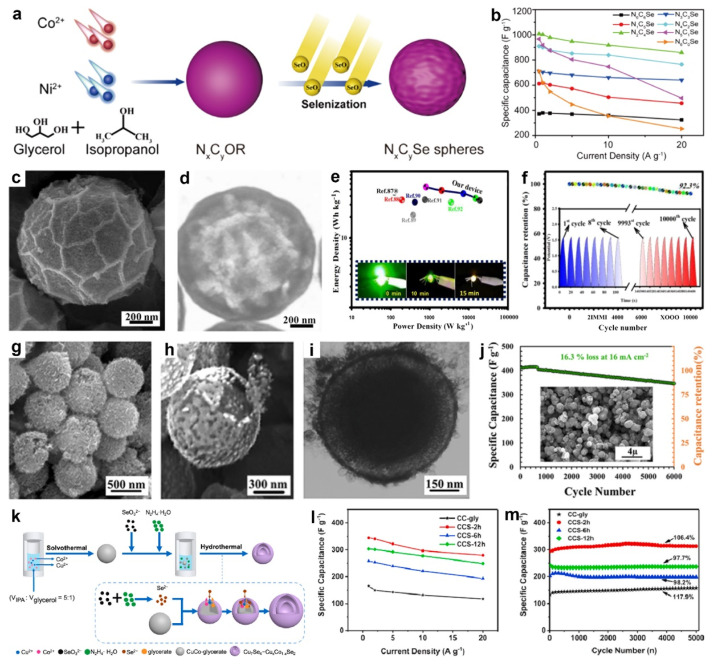
(**a**) Synthesis schematic of N_x_C_y_Se spheres. (**b**) Specific capacitance at different current densities of N_x_C_y_Se spheres. Reproduced with permission [146]. Copyright 2021, American Chemical Society. (**c**) FESEM and (**d**) TEM images of CCSe-HS. (**e**) Ragone plot of the CCSe-HS//AC device. (**f**) Durability performance of CCSe-HS//AC; inset shows GCD traces from cycles 1–8 and 9993–10,000. Reproduced with permission [147]. Copyright 2020, Elsevier. (**g**,**h**) SEM and (**i**) TEM images of yolk-shelled CuCo_2_Se_4_ microspheres. (**j**) Cycling stability of the electrode (inset presents the SEM morphology after cycling). Reproduced with permission [142]. Copyright 2020, American Chemical Society. (**k**) Preparation schematic of copper-cobalt selenide double-shell hollow nanospheres. (**l**) Specific capacitance and (**m**) cycle properties of different samples. Reproduced with permission [110]. Copyright 2020, Elsevier.

In 2021, Zhang et al. synthesized hollow biphase cobalt-nickel perselenide spheres consisting of ~20.86 wt% metastable marcasite-type CoSe_2_ and 79.14 wt% stable pyrite-type NiCoSe_4_ using NiCo-glycerate precursors, with the Ni/Co ratio precisely controlled to tailor the phase composition [146]. As illustrated in Figure 12a**,** Ni(NO_3_)_2_ 6H_2_O and Co(NO_3_)_2_ 6H_2_O were solvothermally reacted in a glycerol/isopropanol mixed solvent to yield NiCo-glycerate with tunable Ni/Co ratios, which were subsequently converted to Co-Ni perselenides (N_x_C_y_Se) via a second solvothermal step. The hollow biphase architecture, coupled with bimetallic synergy, enhanced electron transport and accelerated ion/electron kinetics, affording a specific capacitance of 1008 F g^−1^ at 0.5 A g^−1^ and a high-rate performance of 859 F g^−1^ at 20 A g^−1^ (Figure 12b). Accordingly, an HSC assembled with the N_x_C_y_Se cathode, and a hierarchical porous carbon anode delivered a maximum power density of 7272 W kg^−1^ and a maximum energy density of 34.8 Wh kg^−1^.

Similarly, Zardkhoshoui et al. developed a straightforward self-templated route to copper-cobalt selenide hollow spheres [147]. Specifically, CuCo-glycerate spheres were solvothermally synthesized in isopropanol/glycerol from Cu(NO_3_)_2_ 6H_2_O and Co(NO_3_)_2_ 6H_2_O, then oxidized with KMnO_4_ to yield CuCo_2_O_4_ hollow spheres (CCO-HS). Subsequent dispersion of CCO-HS in ethanol containing Na_2_SeO_3_, followed by dropwise addition of N_2_H_4_ H_2_O under solvothermal conditions, generated Se^2−^ in situ and converted the oxide into CuCo_2_Se_4_ hollow spheres (CCSe-HSs). As shown in Figure 12c,d, CCSe-HSs exhibited wrinkled, nanoparticle-decorated shells and a well-defined hollow, mesoporous interior that provided high surface area and accessible ion-transport channels. The asymmetric device (CCSe-HSs//AC) delivered 53.86 Wh kg^−1^ at 800 W kg^−1^ and reliably lights LEDs (Figure 12e). It also exhibited remarkable durability, retaining ~92.3% of its initial capacitance after 10,000 GCD cycles at 24 A g^−1^, with nearly overlapping first and last eight curves confirming robust stability (Figure 12f).

In a related study, Tavakoli et al. synthesized yolk-shelled CuCo_2_Se_4_ microspheres via a facile hydrothermal route using CuCo-glycerate as the template [142]. During selenization, Se^2−^ first reacted with surface metal ions of CuCo-glycerate, initiating an anion-exchange process that generated a CuCo_2_Se_4_ shell while simultaneous inward Se^2−^ diffusion and faster outward metal-cation diffusion create a core–shell gap (Kirkendall effect). Continued selenization formed a secondary CuCo_2_Se_4_ layer and, upon complete exchange of the core, yielded well-defined yolk-shelled CuCo_2_Se_4_ structures. As shown in Figure 12g,h, the yolk-shelled CuCo_2_Se_4_ microspheres exhibited uniform spherical morphology with an average diameter of ~750 nm and a porous surface composed of densely packed nanoparticles. TEM image (Figure 12i) further revealed distinct contrast between the dark outer shell and the bright inner cavity, confirming the well-defined yolk-shelled hollow architecture. The assembled ASC delivers an energy density of ~9.45 Wh kg^−1^, a power density up to 850 W kg^−1^, and robust cycling stability with only ~16.3% capacitance loss after 6000 charge–discharge cycles (Figure 12j). Similarly, Shirvani et al. synthesized tri-metallic MnNiCoSe yolk-shell spheres confined within nanosheets via a straightforward self-templating route, employing uniform NiCo-glycerate precursors as sacrificial templates followed by a controlled selenization step [148]. Benefiting from abundant redox-active sites, high-surface-area mesoporosity, strong electronic conductivity, and Mn, Ni, Co synergies, the MnNiCoSe electrode delivered 263.67 mAh g^−1^ at 1 A g^−1^, retained 76.63% capacity at 20 A g^−1^, and maintained 84.28% after 10,000 cycles. Moreover, the HSC using MnNiCoSe as the cathode and AC as the anode delivered an energy density of 53.32 Wh kg^−1^ at a power density of 1031.39 W kg^−1^.

In addition, Yang et al. synthesized Cu_7_Se_4_-Cu_x_Co_1−x_Se_2_ double-shell hollow nanospheres via a hydrothermal anion-exchange process using CuCo-glycerate nanospheres as templates [110]. As shown in Figure 12k, CuCo-glycerate nanospheres were first synthesized in a mixed organic solvent and subsequently subjected to a hydrothermal process with Na_2_SeO_3_ as the selenium source and N_2_H_4_ H_2_O as the reducing agent, where SeO_3_^2−^ was in situ reduced to Se^2−^, initiating anion exchange with CuCo-glycerate template to yield Cu_7_Se_4_-Cu_x_Co_1−x_Se_2_ double-shell hollow nanospheres. The resulting Cu_7_Se_4_-Cu_x_Co_1−x_Se_2_ double-shell hollow nanospheres delivered a specific capacitance of 349.1 F g^−1^ at 1 A g^−1^, retained 80.1% of its capacitance at 20 A g^−1^ (Figure 12l), and exhibited outstanding cycling stability with 106.4% retention after 5000 cycles (Figure 12m).

#### 3.2.5. MG-Derived Phosphides

**Figure 13:**
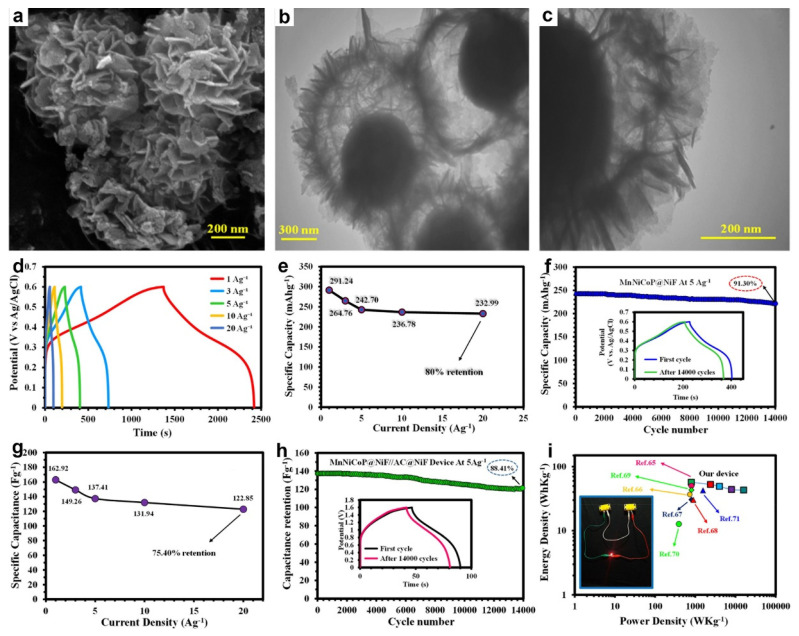
(**a**) FESEM and (**b**,**c**) TEM images of MnNiCoP. (**d**) GCD profiles, (**e**) specific capacitances, and (**f**) cycling stability of the MnNiCoP electrode. (**g**) Specific capacitances, (**h**) durability and (**i**) Ragone plot of the MnNiCoP//AC device. Reproduced with permission [71]. Copyright 2023, Royal Society of Chemistry.

Transition-metal phosphides converted from MG precursors also have emerged as a compelling class of SC electrodes due to their high electrical conductivity and multi-electron Faradaic redox [149,150]. For instance, Shirvani et al. synthesized hollow trimetallic MnNiCoP yolk-shelled spheres composed of interconnected nanosheets via a facile self-templated strategy, employing highly uniform Co-glycerate spheres as sacrificial precursors followed by controlled phosphorization [71]. FETEM (Figure 13a) and TEM (Figure 13b,c) images confirmed a well-defined yolk-shelled hollow architecture, with the spherical shells constructed from closely assembled nanosheets. The hierarchical yolk-shelled hollow architecture with mesoporous channels and high surface area together with the synergistic effect of Mn, Ni, and Co atoms and good electronic conductivity affords markedly enhanced electrochemical performance. Figure 13d confirmed Faradaic behavior in the MnNiCoP electrode. The comparable charge/discharge times across current densities reflect high coulombic efficiency, strong rate performance, and near-reversible Faradaic reactions. MnNiCoP electrode delivered 291.24 mAh g^−1^ at 1 A g^−1^, 80% capacity retention at 20 A g^−1^ (Figure 13e), and long-term stability with 91.3% retention after 14,000 cycles at 5 A g^−1^ (Figure 13f). Moreover, an HSC device (MnNiCoP//AC) showed high specific capacitance of 162.92 F g^−1^ at 1 A g^−1^ and preserved 75.40% of its capacitance at 20 A g^−1^ (Figure 13g). Moreover, the device maintained 88.41% capacity after 14,000 cycles at 5 A g^−1^ (Figure 13h) and achieved an energy density of 57.03 Wh kg^−1^ at ~800 W kg^−1^ (Figure 13i).

#### 3.2.6. MG-Derived Composites

MG-derived composites integrate MG-derived compounds with conductive matrices or secondary active phases to unify fast charge transport with robust, multi-electron redox kinetics [115,134,149]. Exploiting the morphology programmability of MG precursors, these hybrids retain hierarchical porosity while constructing continuous electron- and ion-conduction networks, providing mechanical buffering against volumetric strain and activating rich heterointerfaces [70,73,117]. Broadly, MG-derived composites fall into two categories: first, hybrids that couple MG-derived compounds with conductive media such as carbon materials or conducting polymers, which suppress particle agglomeration, raise accessible surface area, and improve electrical conductivity; second, heterostructures that combine distinct transition-metal compounds, where well-defined interfacial coupling delivers synergistic redox activity and accelerated charge transport.

**Figure 14:**
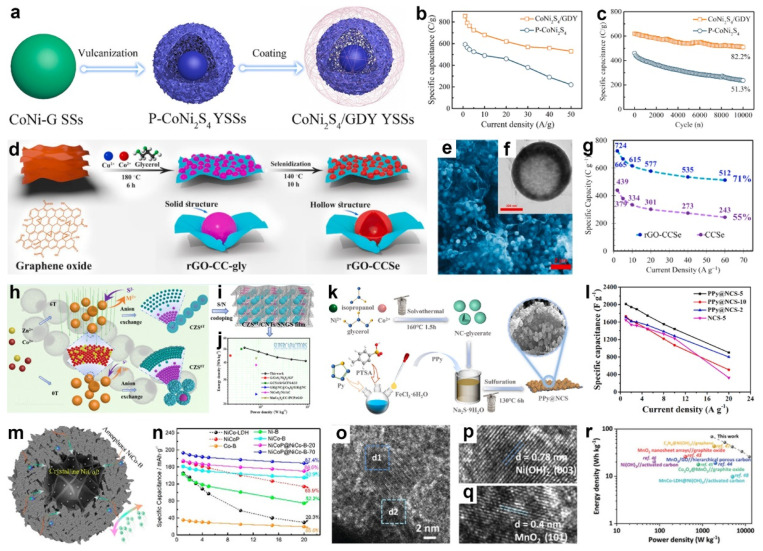
(**a**) Schematic illustration of the preparation for CoNi_2_S_4_/GDY. (**b**) specific capacitance and (**c**) cycling stability of CoNi_2_S_4_/GDY and P-CoNi_2_S_4_. Reproduced with permission [156]. Copyright 2023, Elsevier. (**d**) Schematic representation of the synthesis route to rGO-CCSe. (**e**) SEM and (**f**) TEM images of rGO-CCSe. (**g**) Specific capacitance of rGO-CCSe and CCSe. Reproduced with permission [141]. Copyright 2021, Elsevier. (**h**,**i**) Schematic illustration of the formation of CZS^6T^/CNTs/SNGS. (**j**) Ragone plot of CZS^6T^/CNTs/SNGS//CNTs/SNGS ASC device. Reproduced with permission [157]. Copyright 2020, American Chemical Society. (**k**) Schematic illustration of the preparation of PPy@NCS. (**l**) Specific capacitance of different electrodes. Reproduced with permission [153]. Copyright 2024, Elsevier. (**m**) Ideal microstructures of NiCoP@NiCo-B. (**n**) Specific capacity of different electrodes. Reproduced with permission [116]. Copyright 2024, American Chemical Society. (**o**–**q**) HRTEM images of hierarchical Ni(OH)_2_-MnO_2_ hollow spheres. (**r**) Ragone plots of hierarchical Ni(OH)_2_-MnO_2_ hollow spheres//AC device. Reproduced with permission [158]. Copyright 2022, Royal Society of Chemistry.

Integrating MG-derived transition-metal compounds with conductive components such as reduced graphene oxide (rGO) [124,151], carbon nanotubes (CNTs) [137,152], or conducting polymer [153,154,155] effectively enhanced electrochemical performance. These hybrid structures mitigated particle agglomeration, increased specific surface area, improved electronic conductivity, and buffered structural stress during repeated charge–discharge processes. For instance, Yin and co-workers devised a facile route to a “donor-bridge-acceptor” interface by conformally growing graphdiyne (GDY) on CoNi_2_S_4_ yolk-shell spheres, yielding CoNi_2_S_4_/GDY YSSs [156]. As shown in Figure 14a, CoNi-glycerate solid spheres were first vulcanized to form pristine CoNi_2_S_4_ yolk-shell spheres, followed by in situ GDY deposition. The GDY bridge promoted efficient electron transfer from Ni to Co, generating more interfacial Co^2+^ sites and enabling reversible Co-S/Co-S-OH/Co-S-O redox, thereby boosting capacitance. Simultaneously, the conformal all-carbon layer enhanced structural and interfacial stability, suppressing framework degradation and improving durability. Consequently, CoNi_2_S_4_/GDY YSSs delivered 856 C g^−1^ at 1 A g^−1^ and retained 61.9% (530 C g^−1^) at 50 A g^−1^, versus 27.1% for pristine CoNi_2_S_4_ (Figure 14b), and maintained 82.2% capacitance after 10,000 cycles at 20 A g^−1^, far exceeding the 51.3% of the uncoated counterpart (Figure 14c). Also, the CoNi_2_S_4_/GDY//AC ASC delivered a high energy density of 91.7 Wh kg^−1^ at 793 W kg^−1^, while maintaining 90.8% capacitance after 10,000 charge–discharge cycles.

Moreover, Moosavifard et al. designed a facile self-templated strategy to synthesize porous hollow copper-cobalt selenide microspheres uniformly anchored on conductive networks of reduced graphene oxide (rGO-CCSe) [141]. As outlined in Figure 14d, rGO-CuCo-glycerate precursors were mixed with a selenization solution of ethylene glycol, hydrazine hydrate, and SeO_2_. During the hydrothermal step, hydrazine reduced SeO_2_ to Se^2−^, which converted the Cu/Co precursor into the selenide phase to yield rGO-CCSe. SEM (Figure 14e) and TEM (Figure 14f) images confirmed rGO-CCSe showed a well-defined hollow sphere and firmly anchored on the rGO sheets. The synergistic integration of conductive rGO and hollow porous CCSe architecture provides high surface area and rapid charge pathways, enhancing ion transport, active-site accessibility. Therefore, the rGO-CCSe delivered 724 to 512 C g^−1^ as current density increases from 2 to 60 A g^−1^, outperforming CCSe (439 to 243 C g^−1^) and retaining 71% capacity at 60 A g^−1^ versus 55% for CCSe (Figure 14g). Furthermore, an HSC based on the rGO-CCSe electrode delivered a high energy density of 57.8 Wh kg^−1^ at a power density of 1800 W kg^−1^. In another work, Ye et al. prepared NiCo_2_S_4_@N-CNT via a two-step hydrothermal route, in which hollow NiCo_2_S_4_ nanoparticles (derived from Ni-Co glycerates) were bridged by conductive N-doped carbon nanotubes (N-CNT) [137]. The unique structure afforded a rough, high-area surface, abundant heterointerfaces, and electrolyte-accessible porous channels, enabling rapid ion/electron transport while buffering volume changes during cycling. Benefiting from these features, an all-solid-state asymmetric device (NiCo_2_S_4_@N-CNTs//AC) delivered an energy density of 59.37 Wh kg^−1^ at a power density of 750 W kg^−1^.

In addition, Yu and co-workers synthesized flexible S, N codoped carbon nanotube/graphene film embedded with the one-dimensional (1D) bunched Zn-Co-S yolk-shell spheres (CZS^6T^/CNTs/SNGS) via a high magnetic field-controlled anion-exchange strategy followed by vacuum filtration and one-step S, N codoping/reduction (Figure 14h,i) [157]. The high magnetic field-directed Zn-Co-S yolk-shell balls provided enlarged surface area and porosity, higher crystallinity and conductivity, and accelerated axial ion/electron transport. Integrating these subunits into an S, N-codoped CNT/graphene network suppressed agglomeration and ensures binder-free, intimate contact, thereby increasing redox-active site accessibility, shortening diffusion paths, and enhancing charge transfer. Consequently, the asymmetric device (CZS6T/CNTs/SNGS//CNTs/SNGS) achieved an energy density of 41.1 Wh kg^−1^ at a power density of 9022 W kg^−1^ (Figure 14j).

Yu et al. fabricated a conductive polymer polypyrrole (PPy)-anchored Ni-Co bimetallic sulfide composite (PPy@NCS) as electrode material for hybrid SCs [153]. As shown in Figure 14k, NiCo-glycerate was first synthesized through a one-step solvothermal process, while polypyrrole (PPy) was separately prepared under ice-bath conditions. The resulting NiCo-glycerate was then subjected to a one-pot solvothermal sulfidation in Na_2_S solution, during which in situ polymerization and anchoring of PPy occurred, yielding the PPy@NCS composite. The unique surface is more conducive to exposing more active sites. The PPy@NCS composite couples a conductive PPy network with porous Ni-Co sulfide nanosheets, yielding homogeneous dispersion, strong interfacial bonding, and efficient electron pathways. Its rough, high-surface-area architecture exposed abundant redox sites and accelerated ion transport, while the flexible PPy coating buffered volume changes. Therefore, PPy@NCS-5 delivered a high specific capacitance of 2011.6 F g^−1^ at 1 A g^−1^ (Figure 14l) and maintains 93.3% of its initial capacitance after 5000 cycles at 20 A g^−1^, demonstrating high long-term stability. An HSC assembled with PPy@NCS-5 as the positive electrode and AC as the negative electrode delivered an energy density of 44.6 Wh kg^−1^ at 15,005.6 W kg^−1^.

The coupling of different transition-metal compounds can form well-defined heterojunctions that integrate complementary functionalities. For instance, Shi et al. fabricated crystalline-amorphous core–shell nanospheres (NiCoP@NiCo-B, Figure 14m) by coating nanosheet-assembled NiCoP hollow cores with an amorphous Ni-Co-B shell [116]. Specifically, uniform NiCo-glycerate solid spheres were first prepared by one-pot solvothermal synthesis, then hydrolyzed in deionized water/ethanol to 2D NiCo-LDH. Subsequent NaH_2_PO_2_-mediated phosphidation yielded nanosheet-assembled NiCoP hollow spheres. After redispersion to adsorb Ni^2+^/Co^2+^, an ice-bath NaBH_4_ reduction under N_2_ deposited an amorphous NiCo-B shell, affording crystalline/amorphous NiCoP@NiCo-B core–shell nanospheres. The crystalline NiCoP core ensured mechanical stability, whereas the amorphous NiCo-B shell accelerated electrolyte-ion diffusion; their well-coupled interface furnished abundant redox sites and enabled rapid charge/ion transport. Benefiting from this synergistic design, the optimized electrode delivered a high specific capacity of 193.1 mAh g^−1^ at 1 A g^−1^ and retained 87.4% of its initial capacity at 20 A g^−1^ (Figure 14n), evidencing outstanding rate capability. Moreover, Wei and co-workers synthesized hierarchical Ni(OH)_2_-MnO_2_ hollow spheres via a straightforward template-conversion route [158]. Ni-Mn glycerate solid spheres were first transformed into Ni-Mn hydroxide hollow spheres, followed by O_2_ oxidation to yield Ni(OH)_2_-MnO_2_. The Ni(OH)_2_-MnO_2_ retained their hierarchical hollow architecture. HRTEM (Figure 14o–q) resolved lattice fringes of 0.78 nm and 0.40 nm, indexed to the (003) planes of Ni(OH)_2_ and the (101) planes of MnO_2_, respectively. The resulting architecture offered multiple internal voids, enlarged surface area, and plentiful redox-active sites, facilitating ion/electron transport. An asymmetric solid-state device using a PVA-KOH gel electrolyte, with these hollow spheres as the cathode and activated carbon as the anode, delivered a high energy density of 68.7 Wh kg^−1^ at a power density of 1650 W kg^−1^ (Figure 14r).

## 4. Conclusions and Outlook

In conclusion, this exhaustive review has focused on recent developments in the preparation, design, and supercapacitor application of electrode materials based on metal-glycerates and their derivatives. Their distinct metal ions/glycerolate coordination, compositional tunability, and morphological diversity provide an ideal platform for designing advanced functional materials. Tailored synthetic routes, including solvothermal self-assembly, ion-exchange transformations, and controlled thermal conversions, enable precise construction of solid, hollow, yolk-/multi-shelled, and other multiscale architectures. Subsequent conversion of MGs into functional derivatives (hydroxides, oxides, sulfides, phosphides, selenides, and composites) preserves or amplifies structural hierarchy while introducing complementary electronic structures and redox chemistries.

Despite significant advances (Table 1), several scientific and technological challenges remain before MG-based materials can achieve large-scale implementation in commercial supercapacitors:

(1) Controlled synthesis and structural precision.

Although the effects of metal precursor type/concentration, surfactant dosage, and reaction time on MG morphology have been systematically reported, the complex coordination environment in MGs still demands deeper mechanistic understanding of nucleation and growth to achieve truly uniform morphology, compositional homogeneity, and scalable synthesis. In particular, quantitative control over shell number, inter-shell spacing, defect density, and hierarchical porosity in hollow and yolk-/multi-shelled architectures of MG-based materials remains limited, which hampers rigorous correlation between structure and ion/electron transport behavior. Furthermore, the potential effects of autoclave pressure, temperature, and solvent composition on MG formation have been scarcely explored and thus represent an important direction for future studies on pressure/solvent/temperature modulation in the solvothermal synthesis of MGs.

(2) Interfacial and phase stability

Many MG-derived materials suffer from partial dissolution, phase transformation, volume expansion, or structural degradation under high potentials and high current densities. For sulfides, selenides, and phosphides in particular, redox-driven conversion reactions, interfacial corrosion, dissolution/precipitation, and surface reconstruction can gradually deteriorate active phases and increase internal resistance, yet their detailed cycling degradation pathways remain largely unresolved. Rational interface engineering (e.g., conformal carbon or polymer coatings) and robust heterostructure design are therefore crucial to suppress side reactions, alleviate mechanical stress, and maintain structural integrity during long-term operation.

(3) Mechanistic understanding

Current research on MG-based electrodes is still dominated by “materials fabrication-performance evaluation”, with relatively limited exploration of charge-storage mechanisms, interfacial processes, and dynamic structural evolution. For example, amorphous/crystalline coexisting architectures have been shown to enhance capacitance, yet the underlying synergistic mechanisms (such as defect-mediated redox and internal electric-field effects) remain poorly quantified. Likewise, multi-shelled hollow structures can effectively buffer volume change, but the quantitative relationships among shell number, inter-shell spacing, tortuosity, and ion-diffusion kinetics are not yet established. Future work should therefore place greater emphasis on combining in situ/operando characterization (e.g., XRD, TEM, Raman, XPS, XAS, and synchrotron-based techniques), advanced electrochemical diagnostics (e.g., GITT, EIS modeling, and Dunn analysis), and theoretical simulations (DFT and ab initio molecular dynamics) to elucidate potential-dependent structural evolution, ion/electron transport pathways, and true charge-storage mechanisms in MG-derived systems.

(4) Electrolyte selection and device-level optimization

To date, nearly all reported MG-based and MG-derived supercapacitors operate in aqueous alkaline electrolytes, predominantly KOH solutions or PVA-KOH gels, and systematic studies in non-aqueous or wide-voltage electrolyte systems are, to the best of our knowledge, still lacking. Advancing MG-based devices toward higher energy density without sacrificing power output or durability will therefore require not only sophisticated electrode architectures but also rigorous device engineering. Key directions include rationally matching mass loading and kinetics between positive and negative electrodes, integrating high-voltage aqueous, water-in-salt, or non-aqueous/gel electrolytes with wider electrochemical windows, mitigating the loss of areal capacitance at practical loadings, and ensuring mechanical robustness in flexible or solid-state configurations. It is also crucial to evaluate MG-based devices under realistic operating conditions, such as high areal loading, limited electrolyte, and broad temperature ranges, in order to accurately assess their practical application potential.

## Figures and Tables

**Table 1:** Representative MG precursors and their derivatives as electrode materials for SCs.

**Materials**	**Synthesis Routes**	**Morphology**	**Performances**	**Ref.**
NiMn-glycerate	Solvothermal reaction	Solid sphere	719 C g^−1^ at 1 A g^−1^/rate capability of 60% at 30 A g^−1^/96.5% capacity retention after 10,000 cycles	[78]
NiCo-B-glycerate	Solvothermal reaction and chemical etching	Sphere@nanosheet structure	2036 F g^−1^ at 1 A g^−1^/rate capability of 74.6% at 30 A g^−1^/78.1% capacity retention after 5000 cycles at 10 A g^−1^	[55]
NiCo-LDH	Solution-phase chemical transformations	Nanosheet-assembled hollow spheres	1962 F g^−1^ at 1 A g^−1^/rate capability of 66.4% at 30 A g^−1^/73.3% capacity retention after 3000 cycles at 10 A g^−1^	[105]
Ni-Mn hydroxide	Solution-phase chemical transformations	Hierarchical hollow spheres	1680 F g^−1^ at 2 A g^−1^/1068 F g^−1^ at 15 A g^−1^/96.6% capacity retention after 5500 cycles at 10 A g^−1^	[122]
NiCoZn-carbonate hydroxide	Solution-phase chemical transformations	Flower-like hollow nanospheres	1585.2 F g^−1^ at 1 A g^−1^/rate capability of 72.3% at 10 A g^−1^/87.9% capacity retention after 10,000 cycles at 10 A g^−1^	[72]
NiCo-LDH@MnCo-LDH	Solution-phase chemical transformations	Yolk-shelled spheres	1370.2 F g^−1^ at 1 A g^−1^/1068.2 F g^−1^ at 10 A g^−1^/66% capacity retention after 2000 cycles at 5 A g^−1^	[87]
Hybrid Ni-Mn-Ce oxide	Combined chemical and thermal processes	Hierarchical hollow spheres	688 C g^−1^ at 2 A g^−1^/rate capability of 64.9% at 15 A g^−1^/91.9% capacity retention after 6000 cycles	[69]
NiCo_2_O_4_	Combined chemical and thermal processes	Yolk-shelled spheres	835.7 F g^−1^ at 0.5 A g^−1^/rate capability of 64% at 20 A g^−1^/93% capacity retention after 10,000 cycles at 10 A g^−1^	[133]
Co-Mn mixed oxide	Solution-phase chemical transformations	Double-shelled hollow spheres	860 F g^−1^ at 2 A g^−1^/475 F g^−1^ at 40 A g^−1^/91.8% capacitance retention after 10,000 cycles at 10 A g^−1^	[107]
CuCo_2_O_4_	Thermal treatment strategies	Multi-shelled hollow spheres	691 F g^−1^ at 1 A g^−1^/470 F g^−1^ at 20 A g^−1^/93% capacitance retention after 6000 cycles at 1 A g^−1^	[80]
NiCo_2_S_4_	Solution-phase chemical transformations	Flower-like hollow nanospheres	685.2 C g^−1^ at 1 A g^−1^/rate capability of 66.1% at 30 A g^−1^/73.5% capacity retention after 10,000 cycles at 20 A g^−1^	[139]
CuCo_2_S_4_	Solution-phase chemical transformations	Nanoporous hollow microspheres	1566 F g^−1^ at 2 A g^−1^/rate capability of 59% at 60 A g^−1^/95.7% capacity retention after 5000 cycles at 10 A g^−1^	[140]
Amorphous Ni-Co-Mn sulfide	Solution-phase chemical transformations	Yolk-shelled spheres	1024 C g^−1^ at 2 A g^−1^/608 C g^−1^ at 20 A g^−1^/88.5% capacity retention after 5000 cycles at 10 A g^−1^	[50]
CoS_x_	Combined chemical and thermal processes	Double-shelled hollow spheres	622 C g^−1^ at 2 A g^−1^/rate capability of 74.1% at 20 A g^−1^/74% capacity retention after 10,000 cycles at 5 A g^−1^	[113]
CuCo_2_Se_4_	Solution-phase chemical transformations	Yolk-shelled microspheres	512 F g^−1^ at 1 A g^−1^/rate capability of 70.8% at 6 A g^−1^/83.7% capacity retention after 6000 cycles at 15 A g^−1^	[142]
MnNiCoSe	Solution-phase chemical transformations	Yolk-shelled microspheres	263.67 mA h g^−1^ at 1 A g^−1^/rate capability of 76.63% at 20 A g^−1^/84.28% capacity retention after 10,000 cycles at 10 A g^−1^	[148]
Cu_7_Se_4_-Cu_x_Co_1−x_Se_2_	Solution-phase chemical transformations	Double-shelled hollow spheres	349.1 F g^−1^ at 1 A g^−1^/rate capability of 80.1% at 20 A g^−1^/106.4% capacity retention after 5000 cycles at 10 A g^−1^	[110]
MnNiCoP	Combined chemical and thermal processes	Yolk-shelled spheres	291.24 mA h g^−1^ at 1 A g^−1^/rate capability of 80% at 20 A g^−1^/91.3% capacity retention after 14,000 cycles at 5 A g^−1^	[71]
CoNi_2_S_4_/GDY	Combined chemical and thermal processes	Yolk-shelled spheres	856 C g^−1^ at 1 A g^−1^/rate capability of 61.9% at 50 A g^−1^/82.2% capacity retention after 10,000 cycles at 20 A g^−1^	[156]
rGO-CCSe	Solution-phase chemical transformations	/	724 C g^−1^ at 2 A g^−1^/rate capability of 90% at 60 A g^−1^/91.5% capacity retention after 6000 cycles at 10 A g^−1^	[141]
PPy@NCS	Solution-phase chemical transformations	/	349.1 F g^−1^ at 1 A g^−1^/902.2 F g^−1^ at 20 A g^−1^/93.3% capacity retention after 5000 cycles at 20 A g^−1^	[153]
Ni(OH)_2_-MnO_2_	Combined chemical and thermal processes	Hierarchical hollow spheres	2086.6 F g^−1^ at 2 A g^−1^/rate capability of 46.6% at 20 A g^−1^/88.5% capacitance retention over 5000 cycles at 15 A g^−1^	[158]
NiCoP@NiCo-B	Combined chemical and thermal processes	Core-shelled hollow spheres	193.1 mAh g^−1^ at 1 A⋅g^−1^/rate capability of 87.4% at 20 A g^−1^	[116]

## Data Availability

The datasets used and analyzed in this study are available from the corresponding author upon reasonable request.
